# Differential regulation of mammalian and avian *ATOH1* by E2F1 and its implication for hair cell regeneration in the inner ear

**DOI:** 10.1038/s41598-021-98816-w

**Published:** 2021-09-29

**Authors:** Miriam Gómez-Dorado, Nicolas Daudet, Jonathan E. Gale, Sally J. Dawson

**Affiliations:** UCL Ear Institute, 332 Gray’s Inn Road, London, WC1X 8EE UK

**Keywords:** Cell biology, Molecular biology, Neuroscience, Auditory system

## Abstract

The mammalian inner ear has a limited capacity to regenerate its mechanosensory hair cells. This lack of regenerative capacity underlies the high incidence of age-related hearing loss in humans. In contrast, non-mammalian vertebrates can form new hair cells when damage occurs, a mechanism that depends on re-activation of expression of the pro-hair cell transcription factor *Atoh1*. Here, we show that members of the E2F transcription factor family, known to play a key role in cell cycle progression, regulate the expression of *Atoh1*. E2F1 activates chicken *Atoh1* by directly interacting with a cis-regulatory region distal to the avian *Atoh1* gene. E2F does not activate mouse *Atoh1* gene expression, since this regulatory element is absent in mammals. We also show that E2F1 expression changes dynamically in the chicken auditory epithelium during ototoxic damage and hair cell regeneration. Therefore, we propose a model in which the mitotic regeneration of non-mammalian hair cells is due to E2F1-mediated activation of *Atoh1* expression, a mechanism which has been lost in mammals.

## Introduction

The inner ear consists of specialized sensory organs located in the cochlea and the vestibular system, which are responsible for sound detection and balance respectively. All of these sensory organs contain sensory *hair cells* (HCs), which are mechanoreceptors transducing sound and head movements into signals to the brain via sensory neurons^1^. The HCs are separated from one another and rest upon a basal layer of supporting cells (SCs), which play important roles in the homeostasis and function of the inner ear sensory epithelia^1^. The pro-neural gene *Atoh1*, a basic helix-loop-helix (bHLH) transcription factor, is essential for the formation of inner ear HCs^2^, neural cells in the spinal cord^3^, cerebellar neurons^4^, proprioceptive system neurons^5,6^ and secretory cells in the intestine^7,8^. *Atoh1* mutants in both *Drosophila* and mice lack HCs in all sensory organs, demonstrating the importance of *Atoh1* during neurogenesis and for the differentiation of HC progenitors^2,9–11^.

The loss of HCs is known to underlie deafness and balance disorders. In mammals, the inability to regenerate HCs when they are lost due to the effects of ototoxic agents, noise or ageing is a major cause of sensory hearing loss in humans. This includes the most common form, age-related hearing loss (ARHL) which is highly prevalent in humans with one in three people over the age of 65 having a disabling hearing impairment^12^. ARHL leads to social isolation and has been linked with both depression and dementia^13^. In contrast, non-mammalian vertebrates have a remarkable capacity to regenerate their vestibular and auditory HCs after damage. For example, in the avian auditory epithelium (the basilar papilla), SCs are normally quiescent but can either convert into new HCs or proliferate to form new HCs and SCs after tissue damage^14^. In both types of regenerative responses, *Atoh1* expression is re-activated in SCs before new HCs are produced^14,15^.

The reasons for the limited capacity for HC regeneration in the vestibular organs of mammals and its complete absence in the organ of Corti are unknown, but could be due in part to the inability of adult mammalian SCs to re-activate Atoh1 expression after HC loss. In mammals, including humans, the damage-induced re-activation of *ATOH1*^16^ and the formation of new HCs is very limited^17–20^. Several studies have shown that ectopic overexpression of *Atoh1* resulted in the production of extra HCs at developmental stages^21^ and in young adults^22^. More recently, the overexpression of *Atoh1* in adult human ear tissue has been demonstrated to generate a significant number of cells expressing HC markers^23^. Hence, the potential for a therapeutic approach to hearing restoration based on *Atoh1* is currently being explored in translational research and clinical trials. However, it is likely that other key regulators are missing, since the overexpression of Atoh1 alone, which leads to the production of supernumerary HCs in the immature mammalian inner ear, is not sufficient to trigger the large-scale regeneration of fully differentiated and functional HCs at adult stages. The development of efficient HC regeneration therapies therefore requires a better understanding of the factors acting upstream of, or in conjunction with, Atoh1 to initiate a complete regenerative response in non-mammalian species.

Previous work has shown that the control of mammalian *Atoh1* expression is largely dependent on two downstream cis-regulatory enhancers, known as enhancers A and B^24^. Here, we identified a third highly conserved region in avian species, *enhancer C*, which is not present in mammalian species. Using a combination of bioinformatics analysis, EMSA and reporter gene assays, we identified novel interactions between the *Atoh1* locus and members of the E2F (adenovirus *e*arly gene *2* binding *f*actor) transcription factor family, which are crucial regulators of the cell cycle^25^. We show that E2F1-3 factors have the capacity to up-regulate chick *Atoh1* via direct binding to a novel regulatory element within enhancer C. Furthermore, we observe changes in the nuclear-cytoplasmic localisation of E2F1 during ototoxic-induced HC regeneration that coincides with the re-activation of ATOH1. Given the well-established role of E2F1 in controlling cell cycle re-entry, our findings suggest that enhancer C contributes to the reactivation of Atoh1 expression in mitotic SCs upon HC damage, thereby establishing a mechanistic link between two essential aspects of HC regeneration.

## Results

### Comparative alignment of the avian and mammalian *Atoh1* 3′ sequence and putative binding sites predictions

**Figure 1:**
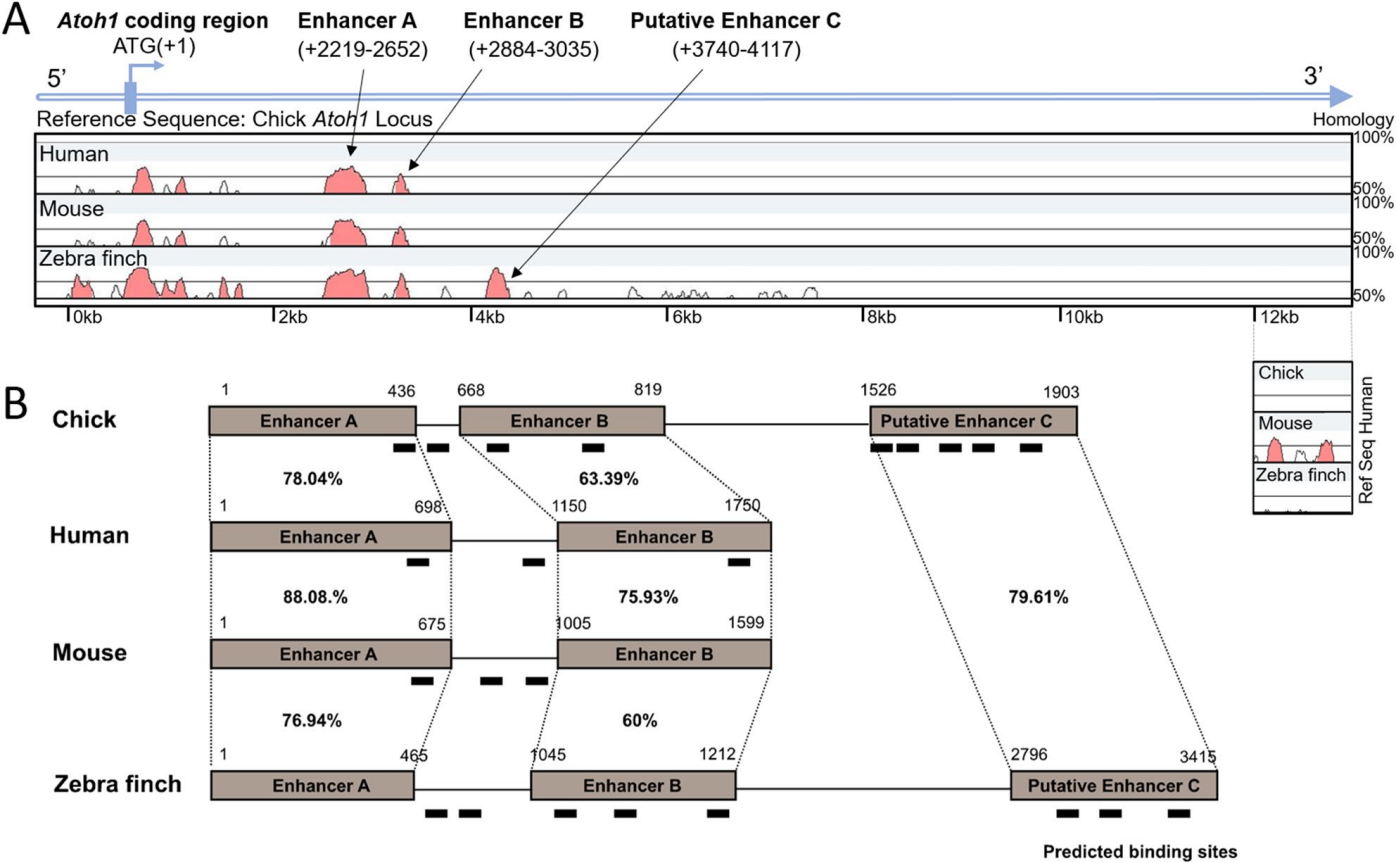
Alignment between the chick, human, mouse and zebra finch *Atoh1* genomic sequences. (**A**) The chick *Atoh1* sequence was used as the basis for comparison and conserved regions are highlighted in pink. Conservation levels are represented in the y axis (in percentages) and the length of the conserved regions is presented in the x axis (minimum length 100 bp). The diagram exported from mVista^27^ shows that some regions within the *Atoh1* coding sequence are highly conserved among chick, human, mouse and zebra finch (*Atoh1* coding sequences correspond to chromosome 6:64729125-64731245 in mouse, chromosome 4:93828753-93830964 in human and chromosome 4: 36493650-36494082 in chick; forward strand, all Ensembl release 91, accessed –December 2017). The two highly conserved regions previously characterized by Helms et al. 2000^24^ and Ebert et al., 2003^28^ and defined as the *Atoh1* enhancer A and B are also conserved among the four species including zebra finch. An additional non-coding region (annotated as putative enhancer C; genomic location 4:37326061-37326437, release 91, updated to 4:37047224-37047600 in latest Ensembl release 102, November 2020; see Supplementary Figure S1 for sequence) shows a high degree of homology between chick and zebra finch but does not share homology with the 3’ region of the mouse and human *Atoh1*. During revision of this manuscript, it was identified that there is some reduced homology between a smaller 268 bp region of the putative chick enhancer C sequence and a sequence several kilobases further downstream of mouse and human Atoh1 genes; its position is shown below the main alignment using the human sequence as the reference. (**B**) Schematic representation (not to scale) of the position of *Atoh1* enhancers A, B and putative enhancer C showing their respective homologies between chick, human, mouse and zebra finch using Clustal2.1. The predicted E2F transcription factor binding sites are marked along the chick, human, mouse and zebra finch *Atoh1* locus.

The regulation of mammalian ATOH1 expression has previously been shown to be controlled by two evolutionary conserved regions (ECRs) downstream of the *Atoh1* locus which are sufficient to drive the expression of an *Atoh1/lacZ* reporter and replicate in most of the tissue specific expression of ATOH1^24,26^. These two regions, defined as the *Atoh1* enhancer A and B are highly evolutionary conserved between mouse and human. In order to identify similarities and differences between *Atoh1* avian and mammalian regulatory regions, a comparative alignment of their sequences was performed. As shown in Fig. 1A, when the human, mouse and zebra finch sequences were aligned to the chicken *Atoh1* 3’ sequence, two highly conserved regions were detected in the four species corresponding to the *Atoh1* enhancers A and B (Fig. 1A). The sequence homology for enhancers A and B across the four species was 81% and 66% respectively (Fig. 1B). Analysing the sequence downstream of these *Atoh1* enhancers, an additional region was found in chicken and zebra finch that shares a similar degree of homology to that of enhancers AB. This is a 377 bp region and lies ~ 700 bp downstream of enhancer B in chick and shares 80% sequence homology between the chick and zebra finch genome. We designated this region as putative enhancer C (genomic location in chick chromosome, originally derived from Ensembl release 91, 4:37326061-37326437; in latest Ensembl release 102, 4:37047224-37047600; for sequence see Supplementary Figure S1).

In order to identify transcription factors potentially playing a role in the regulation of ATOH1 expression, we conducted a bioinformatic analysis using MatInspector software (https://www.genomatix.de/index.html version 9.0, Genomatix, Germany) to identify common transcription factor motifs within enhancers A, B and C from different species (Supplementary Figure S2). One putative transcriptional regulator of *Atoh1* identified by this screen was the E2F transcription factor family. A large number of predicted E2F binding sites were present in the chick, human, mouse and zebra finch *Atoh1* enhancers A and B and additionally within the putative avian enhancer C (Fig. 1B). This included a cluster of 5 E2F sites predicted within the putative enhancer C in chick, with 3 of them specific to the avian species (Fig. 1B). E2F proteins are known to be involved in controlling cell cycle re-entry^25,29–32^ therefore, the predicted E2F binding sites at the *Atoh1* locus were prioritised for further investigation to determine their involvement in linking *Atoh1* re-activation with cell cycle re-entry and cell proliferation.

### E2F1-3 can induce transcription via elements within the chick *Atoh1* but not the mouse *Atoh1* enhancers

**Figure 2:**
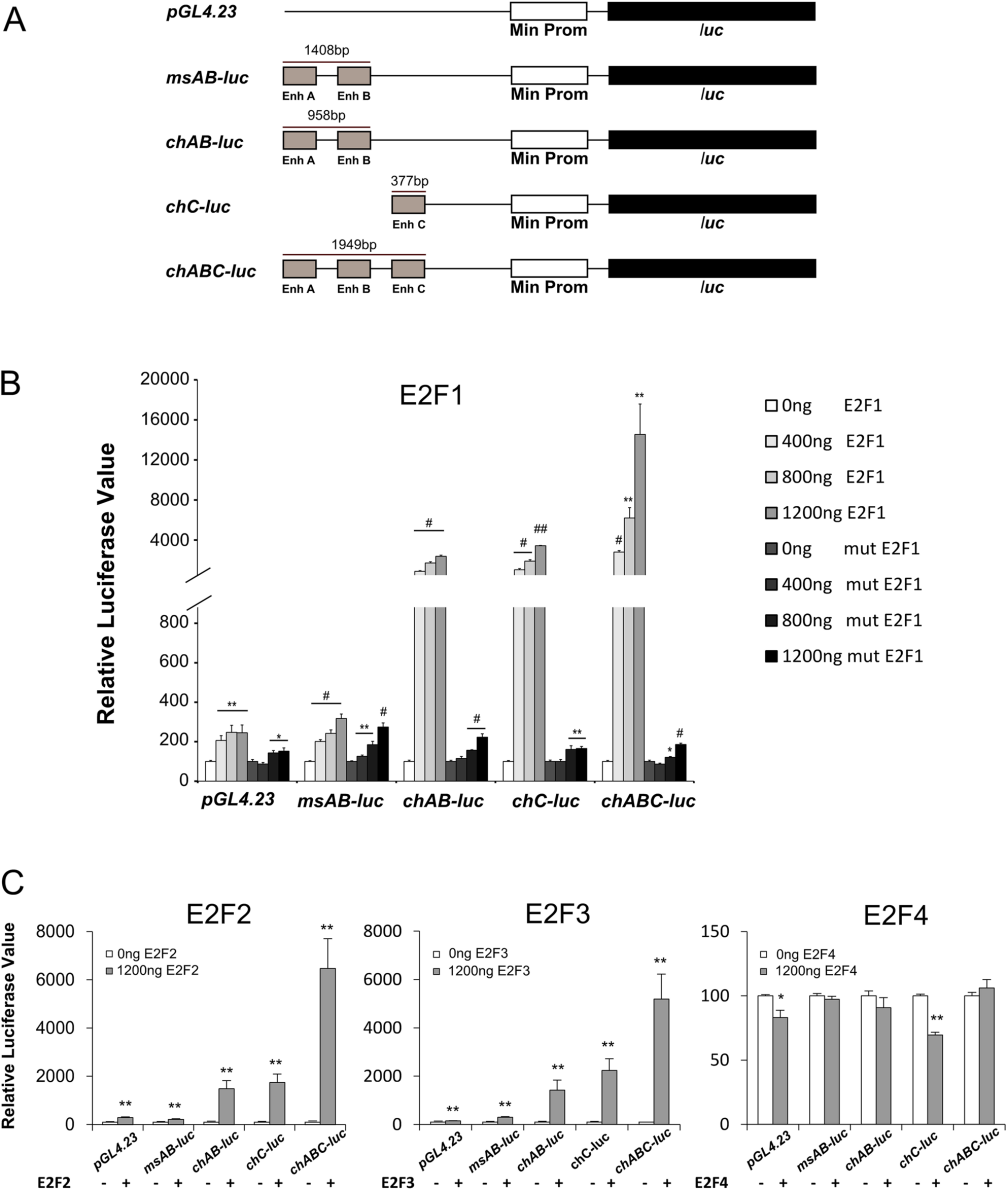
Effect of the E2F transcription factors on the mouse and chick *Atoh1* enhancers and putative enhancer C. (**A**) Schematic representation of the luciferase constructs containing the mouse or chick *Atoh1* conserved regions cloned into the luciferase vector *pGL4.23*. (**B**) Dose–response effects on the *Atoh1* luciferase constructs of transfecting increasing amounts of an E2F1 expression construct or mutant E2F1 in the UB/OC-2 cell line. (**C**) Effect of E2F2, E2F3 and E2F4 on the activity of the *Atoh1* luciferase constructs. Experiments were conducted in triplicate in two separate assays with different DNA preparations for each plasmid in each assay (n = 6). Relative luciferase values are expressed relative to activity of the reporters in the absence of E2F. Student *t*-test was conducted comparing each condition with 0 ng of the corresponding E2F expression construct. (*p < 0.05; **p < 0.01; #*p* < 10^–5^; ##*p* < 10^–10^). Error bars represent the s.e.m (n = 6).

To confirm the validity of the predictions from the bioinformatic analysis, we first tested whether these putative *Atoh1* regulatory elements could confer regulation by E2F1, the primary member of the E2F family, using reporter gene constructs. Distinct mouse and chick *Atoh1* conserved regions containing combinations of the enhancers A, B, or C were cloned upstream of a minimal promoter in luciferase vectors (Fig. 2A). These were then co-transfected with increasing amounts of a human E2F1 expression vector in UB/OC-2 cells, a cell line derived from the immortomouse inner ear epithelium at embryonic day 13^33^. Both *msAB-luc* and *chAB-luc* constructs showed a dose-dependent increase in reporter activity in response to increasing E2F1 levels (Fig. 2B). However, the response of the *chAB-luc* was much larger showing an 8, 17 and 23-fold activation when co-transfected with increasing amounts of E2F1, whereas the maximal response of the *msAB-luc* construct was only threefold. Similarly, the construct containing the *Atoh1* putative enhancer C (*chC-luc*) also showed strong dose dependent activation of 10, 18 and 34-fold difference suggesting that there are functional E2F1 regulatory elements within putative enhancer C. Furthermore, the construct containing all three chick *Atoh1* conserved elements (*chABC-luc*) produced an even greater response: 27, 61 and 145-fold increases in reporter gene activity with increasing amounts of E2F1. To confirm that the activation of the *Atoh1* reporter constructs was dependent on functional E2F1 protein, luciferase co-transfection assays were also performed with a construct expressing a mutant E2F1 protein^34^. When the mutant E2F1 construct was co-transfected, the large up-regulation that was previously observed in the chick *Atoh1* constructs was almost completely abolished, confirming that the activation is dependent on a functional E2F1 protein (Fig. 2B). In contrast, the *msAB-luc* construct showed similar response upon transfection with both mutant or wild type E2F1 expression construct suggesting the small activation observed was not due to a direct interaction of E2F1 with the mouse enhancer. The ability of other members of the E2F family to regulate *Atoh1* constructs was also tested in luciferase assays. As shown in Fig. 2C, E2F2 and E2F3 induced a less pronounced upregulation of the various reporter constructs than E2F1. E2F4, thought to act as a transcriptional repressor^35–38^ did not significantly regulate the mouse or chick *Atoh1* enhancer AB. We did however observe a small, yet statistically significant repression of the putative enhancer C by E2F4 (Fig. 2C).

Taken together, these results indicate that E2F1, E2F2 and E2F3 can strongly active the chick *Atoh1* conserved elements but not the mouse *Atoh1* enhancers. By contrast, E2F4 causes a small downregulation of the putative enhancer C. The greatest activation is observed when the chick *Atoh1* enhancers A and B are combined with the putative enhancer C giving a 145-fold response upon the overexpression of E2F1.

### E2F binds to sequences within the chick *Atoh1* enhancer AB and putative enhancer C

**Figure 3:**
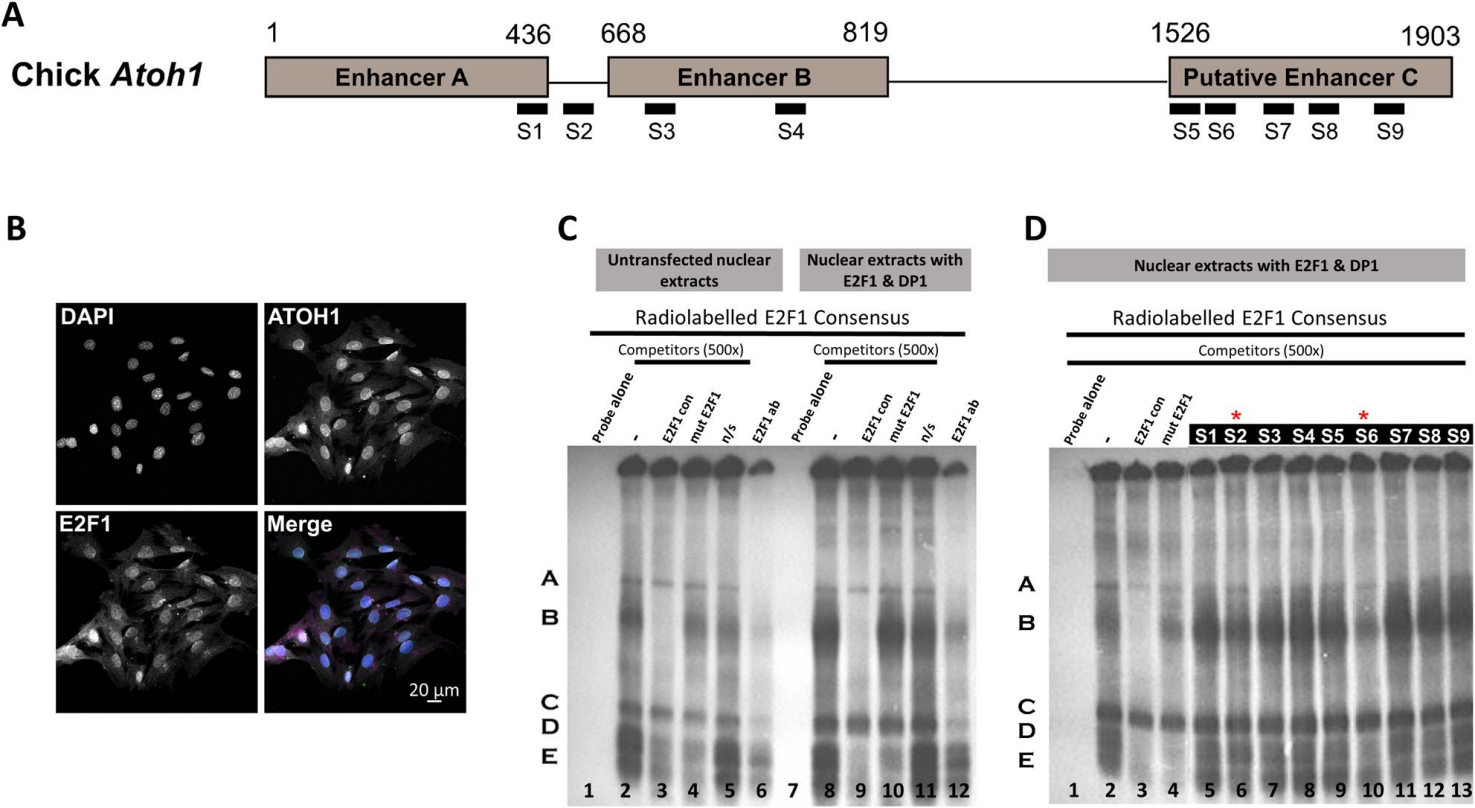
Identification and verification of E2F recognition elements in the chick *Atoh1* conserved elements. (**A**) Schematic diagram showing the location of nine putative E2F binding sites predicted in the chick *Atoh1* conserved regions by MatInspector software (labelled as S1–S9) Numbers indicate the position of the enhancers in base pairs. (**B**) Immunofluoresence detection of endogenous expression of ATOH1 (green) and E2F1 (magenta) proteins in UB/OC-2 cells co-stained with DAPI (blue). (**C**) and (**D**): EMSA analysis performed using a radiolabelled E2F1 consensus sequence, containing a functional E2F1 binding site incubated alone (lane 1), with untransfected nuclear extracts or with nuclear extracts transfected with E2F1 and DP1 expression vectors as indicated. Competition assays were performed with 500 ng of non-radiolabelled competitors (consensus E2F1, lane 3 in both (**C**) and (**D**), and lane 9 in (**C**); mutant E2F1, lane 4 in (**C**) and (**D**), and lane 10 in (**C**); non-specific (N/S) probe, lane 5 and 11 in (**C**); sites S1 to S9 as described in (**A**), lanes 7–15 in (**D**). Lane 6 and 12 show a supershift assay with rabbit polyclonal anti-E2F1, pre-incubated with extracts before the binding assay.

We next tested whether E2F binds directly to any of the nine putative binding sites predicted in the chick *Atoh1* conserved regions (Fig. 3A) using an electrophoresis mobility-shift assay (EMSA). Nuclear extracts from UB/OC-2 cells were used for the analysis of molecular interactions between E2F protein and *Atoh1* sequences since they endogenously express both SC and HC markers^39^ and E2F1 and ATOH1 proteins (Fig. 3B). EMSA experiments with nuclear proteins from UB/OC-2 cells bind to a radiolabelled E2F1 consensus sequence, causing several shifted band complexes (Fig. 3C; lane 2, labelled A–E). Incubating the reaction with a 500-fold excess of an unlabelled consensus E2F1-binding site or a mutated E2F1 consensus sequence identified that bandshift B in particular was attenuated by the E2F1 consensus but not the mutant sequence indicating this shift represents a specific interaction between E2F1 and its consensus binding site (Fig. 3C; lane 3, bandshift B). In agreement with this, EMSA assays using UB/OC-2 extracts transfected with E2F1 and its co-factor DP1^40^ show an increase in intensity of shift B compared with other shifts suggesting this represents the binding to E2F1 protein. Supershift experiments using pre-incubation with an E2F1 antibody appear to attenuate binding of shift B, but also other shifts that are present. To investigate whether any of the chick *Atoh1* E2F binding sites predicted by the bioinformatic analysis can compete with this known E2F binding site, competition was performed by adding a 500-fold excess of the nine unlabelled putative E2F probes predicted in chick *Atoh1* (Fig. 3D; lanes 5–13). None of the nine competitors attenuated binding to the same degree as the consensus E2F1 binding site. However, probe S2 and to a lesser extent probe S6 show some competition in the E2F-specific shift B suggesting that of the nine predicted sites these two are the likeliest E2F binding sites (Fig. 3D; lanes 6 and 10, indicated by red asterisk).

### Activation of the chick *Atoh1* enhancer constructs by E2F1 is dependent on the predicted binding sites 2 and 6

**Figure 4:**
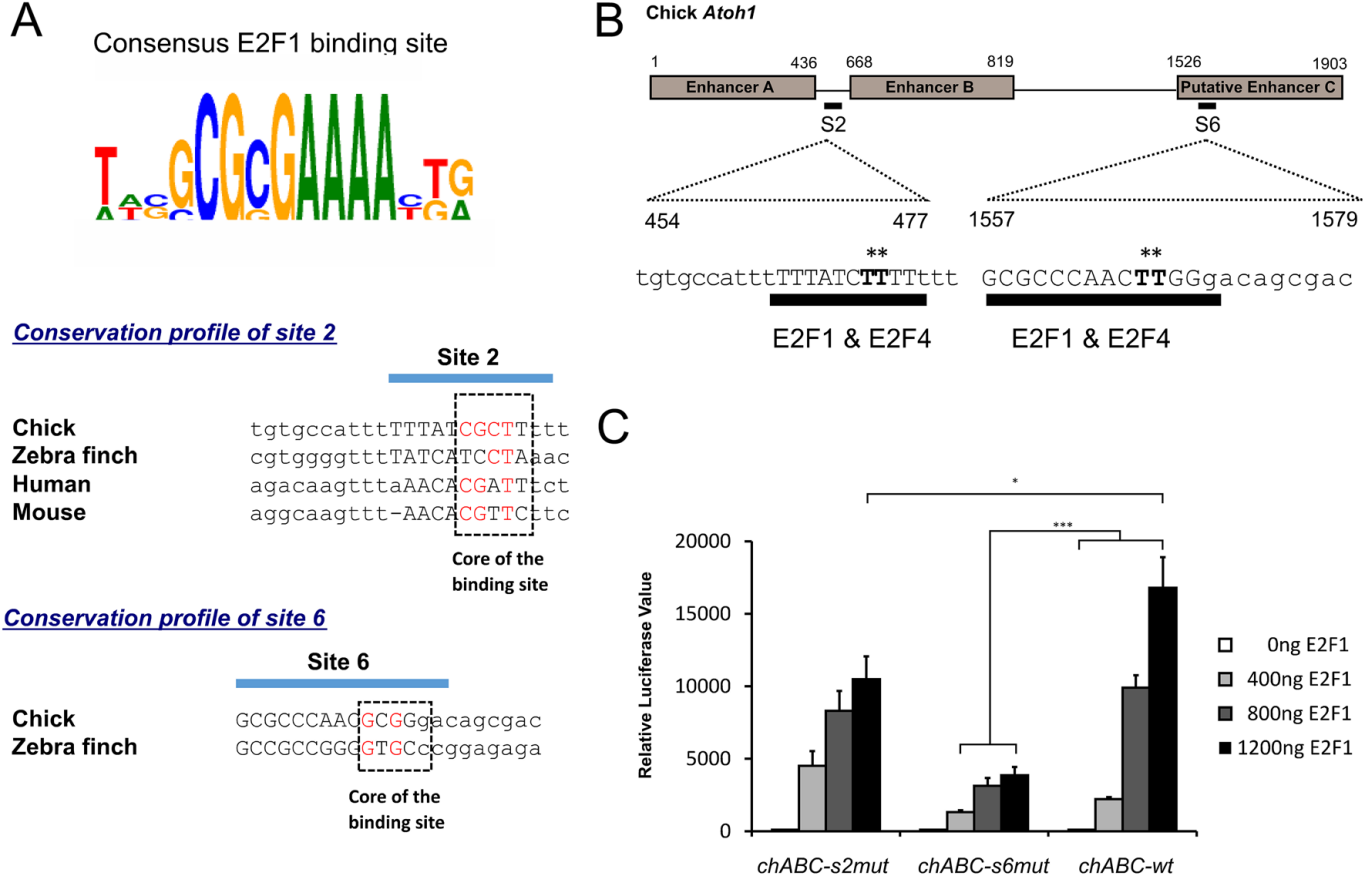
The effect of site directed mutagenesis of the E2F1 putative sites 2 and 6 on E2F1 mediated activation of the *Atoh1* regulatory region. (**A**) Consensus seuqeunce of the E2F1 binding site described by MatInspector, Genomatix. Cross species alignment of sites 2 and site 6 in the *Atoh1* regulatory regions. Conserved nucleotides within the core sequence of site 2 and site 6 are shown in red. (**B**) Diagram showing the sequence and position of putative E2F sites 2 and 6 which both contain overlapping predicted binding sites for E2F1 and E2F4. Point mutations were introduced in the core GC nucleotides, replaced with TT (marked in bold and with asterisks). (**C**) Luciferase assays show a reduced response of the *chABC-s2mut* (*p < 0.05) and the *chABC-s6mut* (***p < 0.001) to E2F1 transfection in comparison with the wild type luciferase construct (*chABC-wt*). Each experiment was conducted in triplicate in two separate assays with different DNA preparations. Student t-test was conducted.

To confirm that predicted E2F sites 2 and 6 identified in EMSA analyses are bound by E2F1 and investigate their role in the activation of the chick *Atoh1* conserved regions we performed site-directed mutagenesis of these sites on the *chABC-luc* construct. Nucleotides to be mutated in site 2 and site 6 were identified by comparing their sequences to the experimentally derived consensus E2F1 binding site^41^ (Fig. 4A). Previous studies have shown that 4 core nucleotides 5’-GCGC-3’ within the E2F consensus sequence are critical for E2F binding activity^42^. We generated two point mutations within this core “GCGC” sequence of site 2 and site 6 in the *chABC-luc* construct (Fig. 4B) to produce *chABC-s2mut* and *chABC-s6mut* luciferase constructs respectively. In the presence of increasing amounts of co-transfected E2F1 the wild type construct (*chABC-wt*) generated a strong and highly significant activation. However, mutation of the putative E2F site 2 (*chABC-s2mut*), attenuated this activation by 37% compared to wild-type sequence. This reduction was observed only when 1200 ng of E2F1 was transfected in comparison to the response given by the wild type construct (Fig. 4C). Whereas, mutation of site 6 (*chABC-s6mut*) caused a greater attenuation, showing a 77% decrease in activation at the highest E2F1 level. The mutation in site 6 within the putative enhancer C also attenuated activation at lower levels of E2F1 transfection; 40% and 68% compared to the wild type construct when 400 and 800 ng of E2F1 were co-transfected respectively (Fig. 4C). These data suggest that both site 2 and site 6 in the chick *Atoh1* flanking sequence play a role in conferring activation of the chick *Atoh1* regulatory regions by E2F1 and that the effect is via a direct interaction between E2F1 and these regulatory elements.

### Profiling changes in E2F1 expression after HC injury in the chick basilar papilla

**Figure 5:**
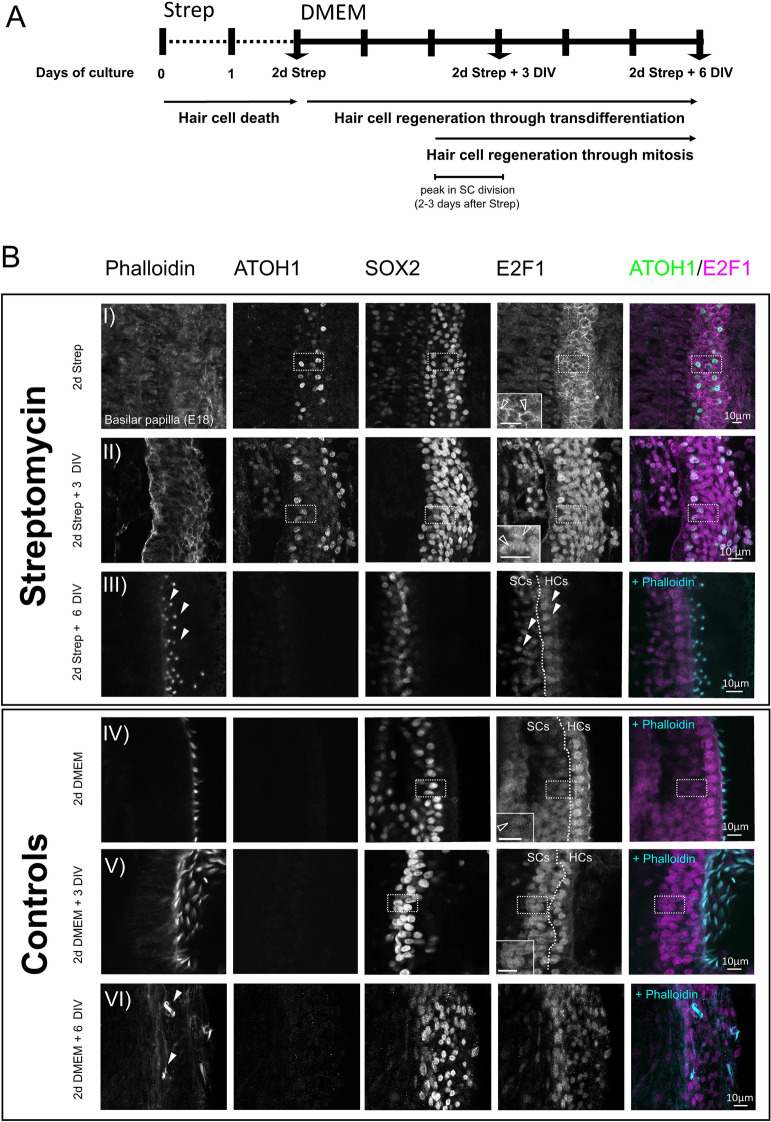
E2F1 expression during HC regeneration in the chick BP. (**A**) Schematic representation of the experimental timeline of organotypic cultures of E18 chick BPs cultured in DMEM and 1% FBS for 2 days with 78 µM streptomycin (Strep) followed an additional 3 or 6 days in vitro (DIV) in the same media without streptomycin. The arrows indicate when the tissue was harvested. (**B**) Immunofluorescence of BPs showing the expression of E2F1 and ATOH1 in HCs (labelled with Phalloidin) and SCs (labelled with SOX2). Cultures maintained for 2 days in media with Strep show ATOH1 re-activation in some SCs whereas a downregulation of nuclear E2F1 expression is observed in comparison to control cultures maintained in DMEM (hollow arrowheads in zoom in areas in row (I) and (IV); scale bar: 10 µm. In BPs cultured for 3 additional days in streptomycin-free media (2d Strep + 3 DIV), nuclear E2F1 expression is observed in SCs (hollow arrowheads in zoom in areas in row II); scale bar: 10 µm). New HCs labelled with Phalloidin are formed in cultures maintained for 6 days in streptomycin-free media after drug treatment (2d Strep + 6 days DIV). In these cultures, ATOH1 is downregulated in SCs labelled with SOX2 whereas E2F1 is expressed in SCs and HCs (arrowheads in row III in E2F1 panel). Control experiments were conducted in parallel in DMEM and at the same timings plus additional days of in vitro (DIV). Technical replicates were three for each experiment with at least three biological samples.

Damaged HCs in the avian inner ear can be replaced via direct conversion of SCs into HCs and through the re-entry of SCs into the cell cycle, which can then give rise to new HCs cells and SCs (reviewed in^43,44^). Given the proliferative functions of the E2F family, we investigated E2F1 expression during the process of HC regeneration using organotypic cultures of the E18 chick basilar papilla (BP) at different time points after HC damage (Fig. 5A). BPs were cultured for 2 days with the ototoxic antibiotic streptomycin, and as expected, a large reduction in the number of HCs was observed (determined by labelling actin-rich hair bundles with phalloidin, Fig. 5B; row I) in comparison to untreated cultures (Fig. 5B; row IV), suggesting that the majority of HCs have been lost. The BP cultures were then maintained for an additional 3 days and 6 days in streptomycin-free media. In cultures allowed to recover for 6 days after drug treatment, new HCs had re-emerged in the BP as shown by phalloidin labelling (Fig. 5B; see arrowheads in row III).

Immediately after 2 days of streptomycin treatment and concurrent with HC loss, ATOH1 expression was up-regulated in SCs (labelled with the support cell marker SOX2 in Fig. 5B; row I) compared to untreated cultures (Fig. 5B, row IV). At this time point, a reduction of nuclear E2F1 as well as an increase in cytoplasmic E2F1 expression was apparent in SCs of treated cultures (Fig. 5B; comparing E2F1 in rows I and IV). After 3 days of recovery, a re-appearance of nuclear E2F1 expression was observed in SCs (Fig. 5B; row II compared to row I). In cultures allowed to recover for 6 days after drug treatment, when new HCs had re-emerged in the BP, ATOH1 expression was downregulated (Fig. 5B; row III). At this time-point, E2F1 was expressed in the nucleus of newly formed HCs and in SCs (Fig. 5; arrows in row III in E2F panel). In control BPs cultured for the same periods of time, but without streptomycin treatment, there were no significant changes in expression or nuclear/cytoplasmic localisation of E2F1 (Fig. 5B; rows IV-VI). However, some HC loss was apparent in BPs that were maintained for the longest period of culture (Fig. 5B; arrows in row VI).

Altogether, these results show that E2F1 is expressed in the HC and SC of the avian BP. During HC regeneration and at the time when ATOH1 expression is reactivated in the damaged BP, the subcellular localisation of E2F1 shifts transiently from the nucleus to the cytoplasm of SCs.

### Enhancer C is well conserved in avian genomes but is not present in non-avian vertebrates

**Figure 6:**
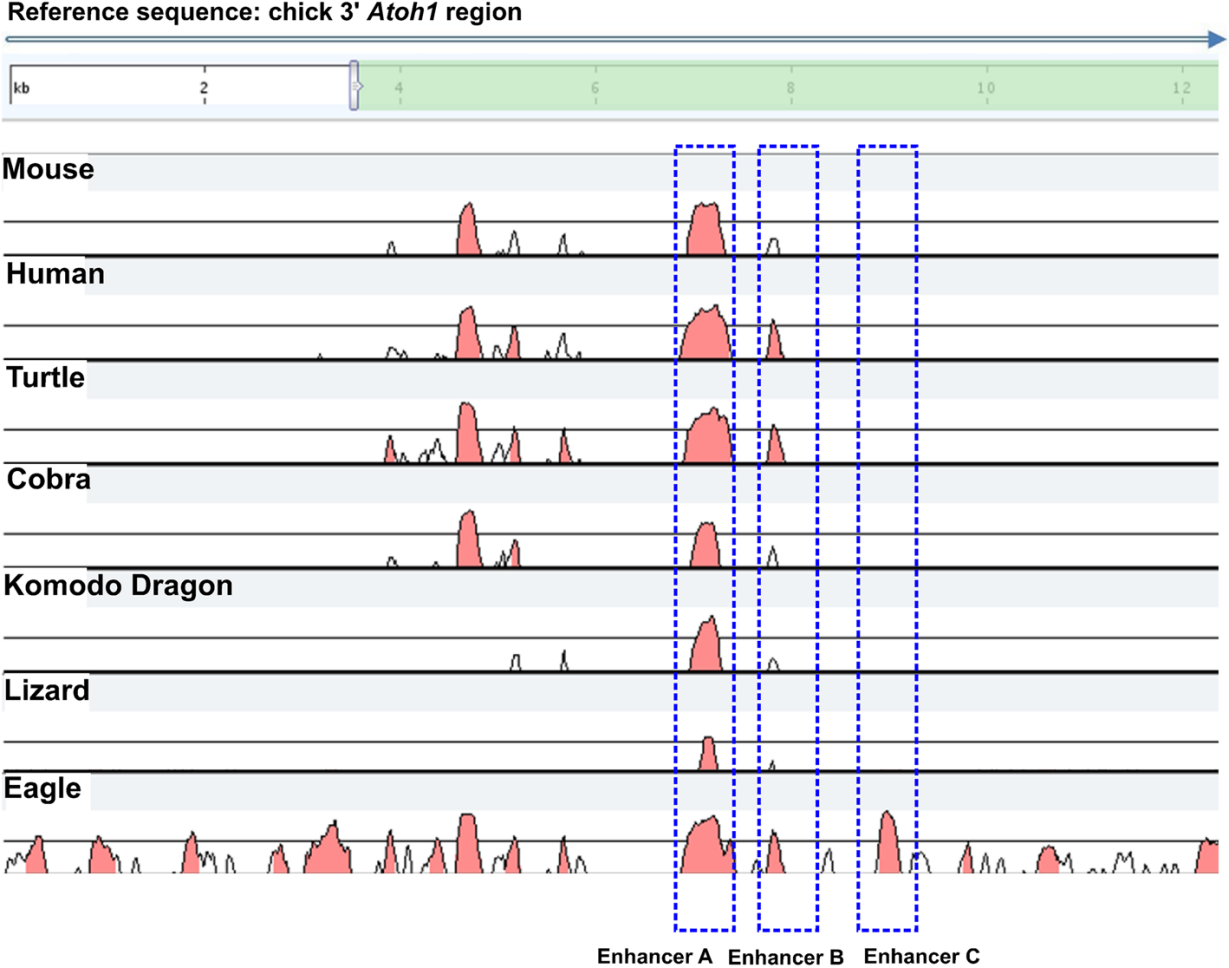
Comparative analysis of the *Atoh1* 3′ sequence in mammalian, avian and non-mammalian non avian species. The comparative alignment was extended to non-avian vertebrate species (Ensembl release 101—August 2020). As shown, the putative enhancer C is not conserved in reptile species but is present in other avian species such as Golden Eagle. A complete analysis of all avian species can be found in Supplementary Data 1.

We investigated how widely the 377 bp chick enhancer sequence (for complete sequence see Supplementary Figure S1) is conserved across other avian species’ genomes and in non-avian vertebrates, some of which can also are able to regenerate hair cells. Figure 6 shows a comparative alignment in a number of species to the chick *Atoh1* locus and reveals that while enhancer A, and to a lesser extent enhancer B, appear to be well conserved in mammals, avians and reptiles, enhancer C is absent from mammals and reptiles. Using Ensembl BLAST to screen all 50 avian, and 21 reptile and amphibian genomes in the Ensembl Release 102 (November 2020) revealed 45 of the 50 avian species’ genomes to have significant conservation of the enhancer C sequence (Supplementary Data S1) whereas no signifcant matches were found to the genomes of reptiles and amphibians. During revision of this manuscript it was identified that there is some homology between a smaller 268 bp region of the chick enhancer C sequence and a sequence several kilobases further downstream of mouse and human *Atoh1* genes (see Supplementary Figure S4). Significantly, this sequence does not include the region of the enhancer C containing binding site S6 that confers E2F1 regulation.

## Discussion

In a comparative analysis of the avian and mammalian *ATOH1* gene locus we found that the sequences of the previously known mammalian 3′ enhancers A and B, which have been shown to be largely responsible for controlling ATOH1 expression^24^, are also conserved in avian species. We also identified a third putative enhancer C, located approximately 700 bp 3′ of enhancer B, the sequence and position of which is highly conserved between chicken and zebra finch. Notably, this 377 bp sequence appears to be absent from both the mouse and human genome (Fig. 1). A subsequent bioinformatic analysis of putative transcription binding sites within these evolutionary conserved regions at the *ATOH1* locus identified multiple putative E2F sites in both mammalian and avian sequences. Further investigation using reporter gene assays and site directed mutagenesis confirmed that sequences within the chicken locus can confer E2F1 regulation upon avian *ATOH1* constructs whereas constructs with mouse enhancers A and B show a similar response to the base vector. All three chicken enhancers, A, B and C, were regulated by E2F1, but site directed mutagenesis indicated that this effect is primarily dependent on an E2F1 binding site within enhancer C (Fig. 4C). A second E2F1 site between chick enhancers A and B, also absent from mammalian sequences, also confers E2F1 activation. These E2F1 responsive sequences are not present in the mouse *Atoh1* locus.

The avian specific regulation of *ATOH1* by E2F1 is intriguing since it provides a potential mechanism by which ATOH1 activation, an event which is required for HC specification might be linked to cell cycle re-entry. In the chick BP, HCs can be regenerated by *direct transdifferentiation* which consists of the phenotypic conversion of SCs into HCs^43,45,46^. In addition, regeneration can also occur via *cell division* of SCs to form new SCs and HCs^14,47–49^. However, the mechanisms controlling cell cycle re-entry during HC regeneration are not well characterised. Hence, one possibility based on our results and the known roles of the E2F family in controlling proliferation is that E2F factors might be involved in the spontaneous regeneration of HCs via cell division and re-activation of *ATOH1* observed in lower vertebrates after damage.

Our results in ex vivo chick BP cultures treated with streptomycin showed that there are dynamic changes in the subcellular localization of E2F1 during HC loss, ATOH1 re-activation and HC regeneration. Soon after HC disappearance, E2F1 is downregulated in the nucleus of SCs and becomes enriched within the cytoplasm and perinuclear region (Fig. 5B-E2F1 expression in row I). Subsequently, a recovery of nuclear E2F1 occurs in SCs 3 days after the end of the streptomycin treatment (Fig. 5B-E2F1 expression in row II). Interestingly, the timing of the re-appearance of nuclear E2F1 expression in SCs is coincidental with the peak of division occurring in vivo during HC regeneration in post-hatch BPs^14,50,51^. A recent transcriptomic study has also demonstrated an up-regulation in E2F1 expression 3 days after ototoxic treatment in the chick BP in vivo^52^, which supports our in vitro results.

**Figure 7:**
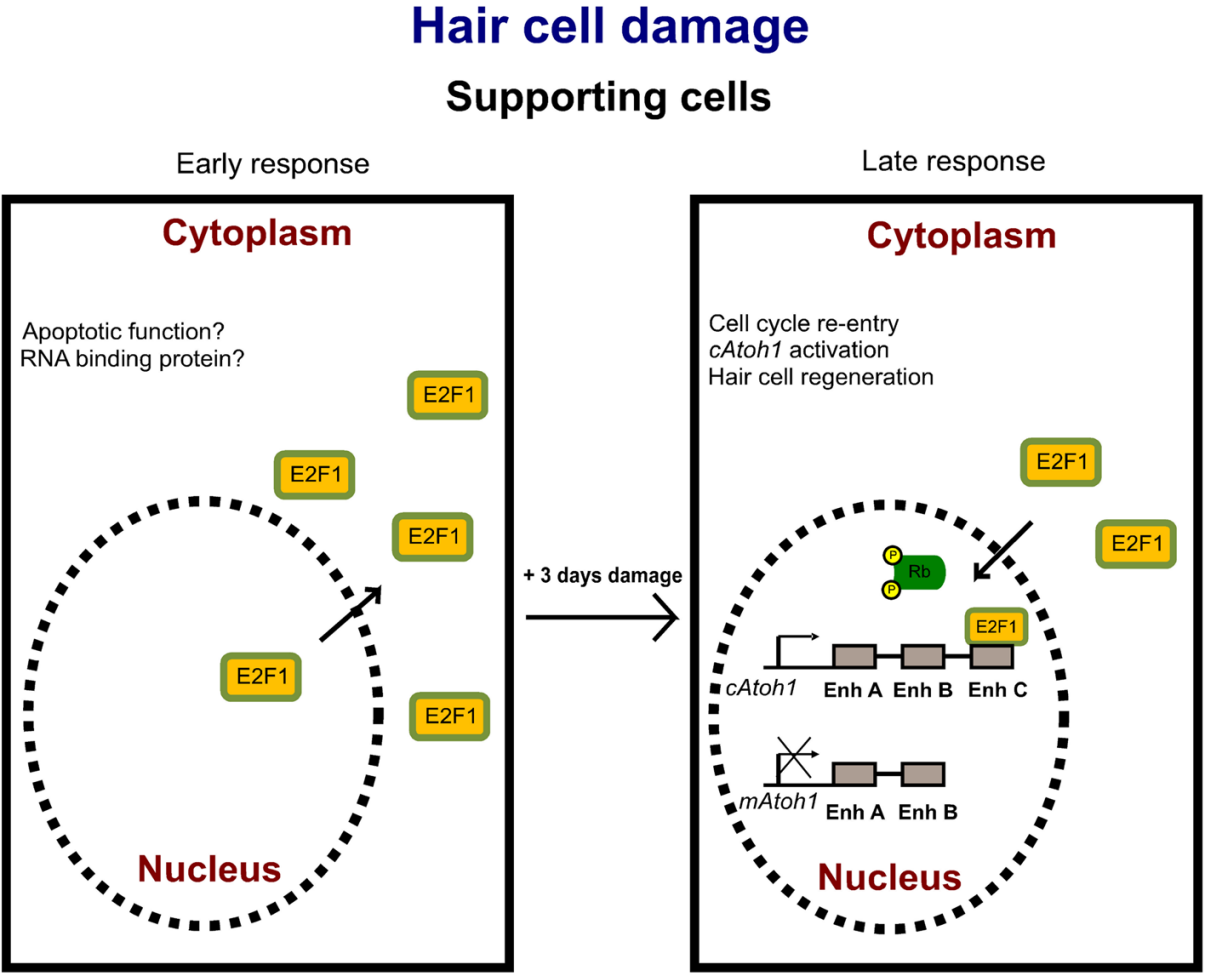
Working hypothesis on the role of E2F1 on the control of *Atoh1*. Upon HC damage, cytoplasmic accumulation of E2F1 in SCs may occur in response to damage as an early response. Cytoplasmic E2F1 may be involved in apoptotic functions or controlling translation of proteins as an RNA-binding protein. A later response might be triggered to activate regeneration via cell division by shifting E2F1 protein into the nucleus of SCs. In the nucleus and upon phosphorylation of the Rb protein, E2F1 promotes re-entry of post-mitotic SCs into the cell cycle (from G_0_ phase to G_1_ phase). Nuclear E2F1 simultaneously induces transcription of chick *Atoh1* via a regulatory element in putative enhancer C resulting in re-activation of the expression of chick ATOH1. This mechanism could be responsible for the spontaneous HC regeneration in avian species. In contrast, mouse *Atoh1* gene is not responsive to E2F1 and therefore limits HC regeneration in mammalian species.

Several publications have reported nucleocytoplasmic shuttling of E2F1 and examined its effect on E2F1 target gene regulation and cell proliferation. In neurons, cytoplasmic E2F1 has been proposed to play a role in regulating apoptosis and/or necrosis^53^. Cytoplasmic E2F1 has also been suggested to control local protein translation as an RNA-binding protein^54,55^. The significance of the nucleo-cytoplasmic shuttling of E2F1 during HC loss and regeneration will require further investigation but it is likely that the cytoplasmic re-localisation of E2F1 prevents it from directly regulating gene expression and controlling cell cycle progression. Alternatively, nuclear E2F1 may be involved in the re-entry of post-mitotic quiescent SCs into the cell cycle. The availability of E2F to mediate gene regulation during the cell cycle is dependent on the Retinoblastoma tumor suppressor (Rb), a protein that belongs to the “pocket proteins” which are phosphorylated/dephosphorylated in response to cyclin-dependent kinases signals^56–58^. Hence, it is possible that nuclear E2F1, although present in mature HCs and SCs in chick, may be sequestered by Rb thereby preventing cell cycle re-entry, *ATOH1* reactivation and HC proliferation. However, upon HC damage, cell signalling could trigger the phosphorylation of Rb and consequently release of E2F proteins leading to the reactivation of avian ATOH1 expression via interaction with enhancer C. Given the requirement for ATOH1 expression in HC specification, this may link SC proliferation with pro-HC gene expression in avians as proposed in Fig. 7. Further work is required to substantiate these hypotheses and to understand what role E2F re-localisation might play in this process.

Studies with E2F knockout mice suggest unique tissue-specific roles for each E2F member during mouse development^59^. However, the phenotype in the inner ear in single and compound knockout animals has not yet been examined^59,60^. Studies with Rb mutant mouse models have examined the phenotype in the inner ear and demonstrated that there is an abnormal proliferation of vestibular and cochlear HCs during development^61–64^. This might be mediated by the effect of unbound E2Fs stimulating the cell cycle in the absence of Rb.

In conclusion, our data provide new insight into the comparative regulation of the key transcription factor *ATOH1* in mammals and avians. Analysis of the genomes of all completed avian, reptile and amphibian species confirm the conservation of the 377 bp enhancer C sequence is avian specific whereas *Atoh1* enhancers A and B is conserved across these species. Our results emphasize the potential role that a novel non-coding regulatory region, enhancer C, might play during avian HC regeneration to coordinate cell cycle re-entry with the re-activation of *ATOH1* expression. Therefore, it may also have relevance to therapeutic approaches to preserve human hearing through potential strategies designed to modify the mammalian expression of *ATOH1*.

## Material and methods

### Bioinformatic analysis

Evolutionary conserved regions in the genomic sequence flanking the *Atoh1* gene were identified using the comparative genomics browser mVista. Clustal2.1 was used to calculate the percentage homologies among the chick, human, mouse and zebra finch *Atoh1* genomic sequences. MatInspector was used to predict transcription binding sites [Genomatix Platform; https://www.genomatix.de/index.html^65,66^]. Nine predicted E2F binding sites (S1–S9) were predicted within the enhancer regions and selected for functional analysis.

### Plasmids and constructs

All luciferase constructs contain the *Atoh1* conserved regions upstream of the minimal promoter and were made using pGL4.23 [luc2/minP] luciferase vector (Promega). The *msAB-luc* and *chAB-luc* were constructed by digesting a J2XnGFP containing the mouse *Atoh1* enhancer (1408 bp) and a PE1-chick homology-AB vector containing the chick *Atoh1* enhancer (1015 bp), kindly provided by Professor Jane Johnson (University of Texas Southwestern Medical School, USA) and subcloned into the pGL4.23[luc2/minP] luciferase vector. The *chC-luc* (genomic location in chick chromosome genomic location in chick chromosome 4:37047224-37047600; Ensembl release 102 –November 2020) was made by amplifying chicken genomic DNA using the following primers: Chick C-forward, 5′-TAAGCAGTCGACTGTCCTCTC-GCCCGCCCTG-3′; Chick C-reverse, 5′-ATTCGTGTCGACGGTTACAGTGTCGGTGAGCTGC-3′. The amplified PCR product (383 bp) was cloned into the pGL4.23 [luc2/minP] luciferase vector. The *chABC-luc* was generated by amplifying chick genomic DNA with the primer Chick ABC-forward: 5′-TAGAAGTCGACGCAGCGCATTTCCATGTTGAG-3′ and the Chick C-reverse listed above. After PCR amplification, the ABC fragment (1905 bp) was cloned into the pGL4.23 [luc2/minP] luciferase vector. The following expression constructs used in transfections have been previously reported: a human E2F1 expression construct cloned in pcDNA3 (kindly obtained from Professor Kristian Helin, University of Copenhagen, Denmark^67^, mutant E2F1 containing a change in the DNA binding domain located in exon 3 in the E2F1 cDNA^34^, E2F2 is a pCMV-Neo-Bam construct^68^, E2F3 is a pcDNA3 construct^69^, E2F4 is a pcDNA3 construct^70^ and human DP1 cDNA (accession number: L23959) is a pCMV-Neo-Bam1 (a gift from Dr Tony Kouzarides, The Gurdon Institute, University of Cambridge, UK) subcloned into the pcDNA3 expression construct.

### Transient transfections and luciferase activity assays

For Dual Luciferase Assays, UB/OC-2 cells were cultured in MEM media supplemented with 10% FBS and 50 U/ml γ-IFN at 33 °C^39^. Prior to transfection, cells were plated in 6 well plates (2 × 10^5^ cells per well) at 37 °C and transfected using the calcium chloride method^71^ with 200 ng of the *Atoh1* reporter constructs with increasing amounts of E2F expression constructs (as indicated in Fig. 2) and 10 ng of phRL-null (Promega). The total amount of transfected DNA was maintained constant using the empty pSI mammalian expression vector (Promega). A glycerol shock was conducted 24 h after transfecting the DNA reporter constructs and then cells were cultured for 16 h in complete media at 33 °C. Following this, cells were harvested and enhancer activity was quantified using the Dual Luciferase Reporter Assay System (Promega). Each assay was conducted in triplicate in two separate experiments using different DNA preparations for each plasmid.

### Electrophoretic mobility-shift assays (EMSA)

EMSA experiments were performed with nuclear extracts from UB/OC-2 cells transfected with E2F1 and DP1 (the co-factor for E2F) since a significant improvement in the E2F-DP1 complex interacting with a consensus E2F binding site was found (Fig. 3C). Double stranded oligonucleotides probes (for sequences see Supplementary Table S1) were labelled in a standard T4 kinase reaction with γ^32^P γATP (GE Healthcare). Binding reactions were conducted at room temperature for 10 min with 10 µl of 2 × Parker buffer (16% Ficoll, 40 mM HEPES at pH 7.9, 100 mM KCl, 2 mM EDTA, 1 mM DTT, 4 mM MgCl_2_), 1.5 µg poly (dI·dC), 10ug of nuclear extracts protein and 50 ng of the radiolabelled oligonucleotide. For competition experiments, an excess of 500-fold molar of cold consensus or mutant oligonucleotide competitors were added to the reaction for 2 h at room temperature before adding the radiolabelled oligonucleotide. For supershift experiments, 1 µg of antibody was added to the binding reaction 1 h before adding the radiolabelled probe. Reaction mixtures were resolved on a 4% poly-acrylamide gel (29:1) 0.25 × TBE acrylamide gel and electrophoresed in 0.25 × TBE at 200 V for 2 to 3 h at 4 °C. The polyacrylamide gel was dried under a vacuum system for 1–2 h at 80 °C and exposed to an X-ray film at − 80 °C for between 10 h and 5 days. Following film exposure, film was developed using Kodak film reagents according to the manufacturer’s instructions.

### Site-directed mutagenesis

The *chABC-s2mut* and *chABC-s6mut* luciferase constructs were generated by QuickChange II Site-Directed Mutagenesis (Agilent Technologies). Oligonucleotides were designed using the primer design tool provided on the manufacture’s website (http://www.genomics.agilent.com/primerDesignProgram.jsp) to introduce two point mutations in the predicted E2F site 2 and site 6 in the *chABC-luc* construct. Primers designed for this technique were (mutated bases underlined): P2_Sense_mutagenic primer, 5′-CGCTTTAAAGAAA-TGCCTCAAAAAAAGATAAAAAATGGCACAAAGCAAAGC-3′, P2_Antisense_mutagenic primer, 5′-GCTTTGCTTTGTGCCATTTTTTATCTTTTTTTGAGGCATTTCTTTAAAGCG-3′, P6_Sense_ mutagenic primer, 5′-TCCCGCGCCCAACTTGGGACAGCGACGC-3′, P6_Antisense_mutagenic primer, GCGTCGCTGTCCCAAGTTGGGCGCGGGA.

### Immunohistochemistry

Immunohistochemistry on chick tissues was performed as described previously^72^ with the following antibodies: chicken anti-peptide polyclonal against mouse *Atoh1* (used at 1:5000) kindly supplied by Matthew Kelley^73^; rabbit polyclonal E2F1 (against the N terminal of E2F1; P100821_P050) from Aviva Systems Biology used at 1:200; mouse monoclonal anti-SOX2 (BD Pharmingen) used at 1:200. Secondary antibodies (Invitrogen) were: goat anti-(rabbit Ig) conjugated to Alexa Fluor 633 (1:1000), goat anti-(mouse Ig) conjugated to Alexa 550 (1:1000) and goat anti-(chick IgY) conjugated to Alexa 488 (1:1000). Samples were counterstained with Phalloidin 405 (1:1000) and imaged on a Zeiss LSM Meta 510 and 880 confocal microscopes (Zeiss). For cell line immunohistochemisty, UB/OC-2 cells were seeded in 6 well plates containing 13 mm diameter uncoated glass coverslips (VWR). Cells were grown until 80% confluency and fixed in 4% paraformaldehyde for 2 min. Following fixation, cells were washed three times in PBS for 5 min before proceeding for immunohistochemistry with ATOH1 and E2F1 antibodies as described above.

### Explant cultures

Basilar papillae (BPs) were dissected from embryonic chicks at E18 as described previously^51^ and maintained in chilled Leibovitz’s L-15 media (Gibco, Invitrogen) during the dissecting procedure. Isolated BPs were cultured onto Millicell cell culture inserts (Millipore) placed into 35 mm culture dishes containing 1.5 ml of Dulbecco's Modified Eagle Medium (DMEM) supplemented with 1% fetal bovine serum and 1 mM HEPES buffer. For the drug experiment, 78 μM of streptomycin (Sigma) was added to the previous culture medium. Cultures were maintained for 2 days in vitro (DIV) at 37 °C and 5% CO_2_. After 2 days of incubation with streptomycin, all BPs were either harvested or were rinsed and maintained in culture for 3 or 6 days in streptomycin-free media which was replenished at 2 days intervals (see Fig. 5A). Untreated cultures were maintained in parallel and served as matched controls. For each experiment, three biological replicates were performed with a minimum of 3 samples per run. All animal procedures were carried out in accordance with the UK Home Office guidelines and approved by University College London and by the UK Home Office.

## Supplementary Information

Supplementary Information.

Supplementary Figure S1.

Supplementary Figure S2.

Supplementary Figure S3.

Supplementary Figure S4.

Supplementary Table S1.

## Data Availability

All data generated or analysed during this study are included in this published article (and its Supplementary Information files).
