# Supplementary material for: Differential regulation of mammalian and avian ATOH1 by E2F1 and its implication for hair cell regeneration in the inner ear
Source: Sci Rep. 2021 Sep 29;11:19368. doi: 10.1038/s41598-021-98816-w (PMC8481459; doi:10.1038/s41598-021-98816-w)
Supplement: Supplementary file 2 — Supplementary Figure S1. [file 41598_2021_98816_MOESM2_ESM.pdf]

Differential regulation of mammalian and avian *ATOH1* by E2F1 and its implication for hair cell regeneration in the inner ear

Miriam Gómez-Dorado[1], Nicolas Daudet[1], Jonathan E. Gale[1], Sally J. Dawson[1]*

[1]UCL Ear Institute, 332 Gray's Inn Road, London WC1X 8EE, UK

Supplementary Figure S1. Sequence of 377bp chick enhancer C region.

Ensembl release 91, December 2017

>Build 2017: 4 dna:chromosome chromosome:Gallus_gallus-5.0:4:37326061:37326437:1

TGTCCTCTCGCCCGCCCTGGTGCGCGCTCCCGCGCCCAACGCGGGACAGCGACGCGCACC

CGAGCGGTGCTGCCGCGCTTTATGGAGCGGTTAATCAACTGCGCATCAGCGAGACAGCGC

ATCAGCCCATCTGCTTGATATATATTCAGGAGGGCTCCAGCCCTTTTGAAGTCTAATTTC

TTCCCCGGGAGAACGCGCCGGGTAATTTACCATCATTTCATACGCATCGCGGCCACCGAG

TTAACCCTTTCCCCAAGCGCTGCCCGCACGGGATTTTCCTCCCGAAAAACGCCGGGTGCA

ACGCTACAAACTTGTCAAAGCGTCTTTCCGATGATGCTCCTTGAAATAAGAGATCGCAGC

TCACCGACACTGTAACC
