# Supplementary material for: Differential regulation of mammalian and avian ATOH1 by E2F1 and its implication for hair cell regeneration in the inner ear
Source: Sci Rep. 2021 Sep 29;11:19368. doi: 10.1038/s41598-021-98816-w (PMC8481459; doi:10.1038/s41598-021-98816-w)
Supplement: Supplementary file 3 — Supplementary Figure S2. [file 41598_2021_98816_MOESM3_ESM.pdf]

Differential regulation of mammalian and avian *ATOH1* by E2F1 and its implication for hair cell regeneration in the inner ear

Miriam Gómez-Dorado[1], Nicolas Daudet[1], Jonathan E. Gale[1], Sally J. Dawson[1]*

[1]UCL Ear Institute, 332 Gray's Inn Road, London WC1X 8EE, UK

Supplementary Figure 2. Transcription Factor Motif Analysis of *Atoh1* enhancers A, B and C.

Legend: MatInspector predictions for transcription factor motifs common to human, mouse, chick and zebra finch *(a-c),* or common to enhancer C of chick and zebra finch, *(d)* . The family names are given as a unique identifier for each match and displayed with a first letter corresponding to the section family they belong to (e.g. vertebrates (V$), other (O$). Each matrix match is represented by a coloured half round symbol with can be found in the positive or negative strand. Additionally, matrix family are presented in a list at the bottom of the figure. The matrix similarity is a parameter established by MatInspector showing how similar a prediction is in comparison to the consensus matrix:

a) Common transcription factor binding sites predicted within the *Atoh1* enhancer A in human, mouse, chick and zebra finch. A total number of 52 common transcription factor families matches were predicted on enhancer A.

b) Common transcription factor binding sites predicted within the *Atoh1* enhancer B in human, mouse, chick and zebra finch. A total number of 62 common transcription factor families matches were predicted on enhancer B.

c) A V$NEUR matrix family match was predicted for binding the mouse and human *Atoh1* enhancer B. The sequence of the predicted V$NEUR matrix family matched an E-box sequence which is the functional binding site for ATOH1. Other matrix families with roles in cell fate, differentiation and maturation are predicted for binding the *Atoh1* enhancers. These include the POU3 and POU4 transcription factors (Family V$BRNF), BARHL1 (Family V$HOMF), GATA (Family V$GATA), LHXF (Family V$LHXF), the Id transcription factors (Family V$NEUR), YY1 (Family V$YY1) and E2F (Family V$E2FF) (marked in red boxes in the list of matrix families).

d) Common transcription factor binding sites predicted within *Atoh1* putative enhancer C in chick and zebra finch. A total number of 53 common transcription factor family matches were predicted on putative enhancer C in chick and zebra finch. Some of the family matrices found in the putative enhancer C were also predicted for binding the *Atoh1* enhancer A and enhancer B. Some examples were marked in red squares such as the V$NEUR, V$E2F and V$NFKB matrix families.

a)

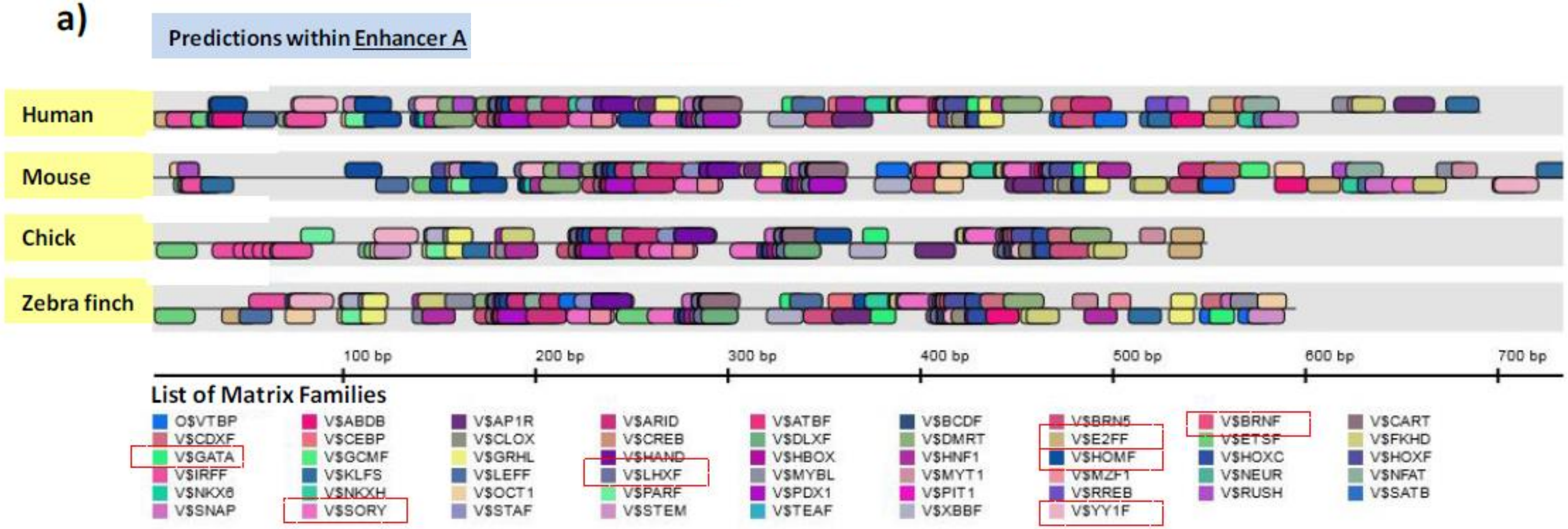

Predictions within Enhancer A
Human
Mouse
Chick
Zebra finch
100 bp
200 bp
300 bp
400 bp
500 bp
600 bp
700 bp
List of Matrix Families
O$VTBP
V$CDXF
V$GATA
V$IRFF
V$NKX6
V$SNAP
V$ABDB
V$CEBP
V$GCMF
V$KLFS
V$NKXH
V$SORY
V$AP1R
V$CLOX
V$GRHL
V$LEFF
V$OCT1
V$STAF
V$ARID
V$CREB
V$HAND
V$LHXF
V$PARF
V$STEM
V$ATBF
V$DLXF
V$HBOX
V$MYBL
V$PDX1
V$TEAF
V$BCDF
V$DMRT
V$HNF1
V$MYT1
V$PIT1
V$XBBF
V$BRN5
V$E2FF
V$HOMF
V$MZF1
V$RREB
V$YY1F
V$BRNF
V$ETSF
V$HOXC
V$NEUR
V$RUSH
V$CART
V$FKHD
V$HOXF
V$NFAT
V$SATB

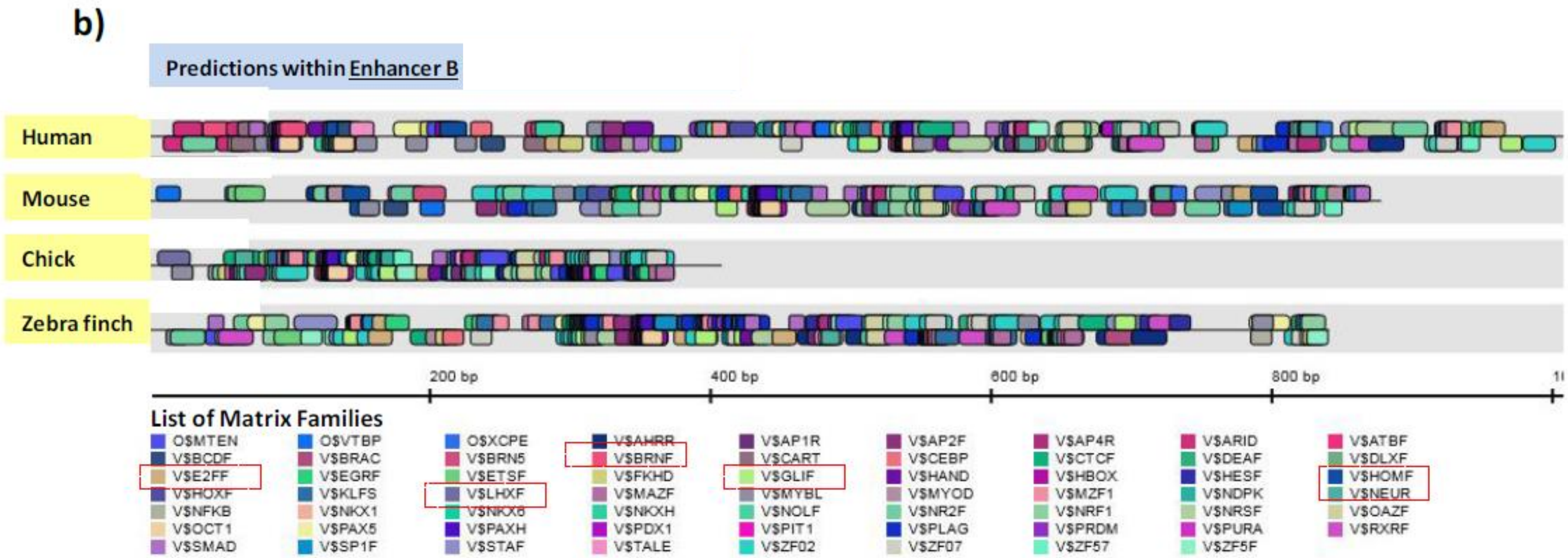

b)
Predictions within Enhancer B
Human
Mouse
Chick
Zebra finch
200 bp
400 bp
600 bp
800 bp
List of Matrix Families
O$MTEN
O$VTBP
O$XCPE
V$AHRR
V$AP1R
V$AP2F
V$AP4R
V$ARID
V$ATBF
V$BCDF
V$BRAC
V$BRN5
V$BRNF
V$CART
V$CEBP
V$CTCF
V$DEAF
V$DLXF
V$E2FF
V$EGRF
V$ETSF
V$FKHD
V$GLIF
V$HAND
V$HBOX
V$HESF
V$HOMF
V$HOXF
V$KLFS
V$LHXF
V$MAZF
V$MYBL
V$MYOD
V$MZF1
V$NDPK
V$NEUR
V$NFKB
V$NKX1
V$NKX6
V$NKXH
V$NOLF
V$NR2F
V$NRF1
V$NRSF
V$OAZF
V$OCT1
V$PAX5
V$PAXH
V$PDX1
V$PIT1
V$PLAG
V$PRDM
V$PURA
V$RXRF
V$SMAD
V$SP1F
V$STAF
V$TALE
V$ZF02
V$ZF07
V$ZF57
V$ZF5F

c)

tcccCAGCTGcgc

V$NEUR

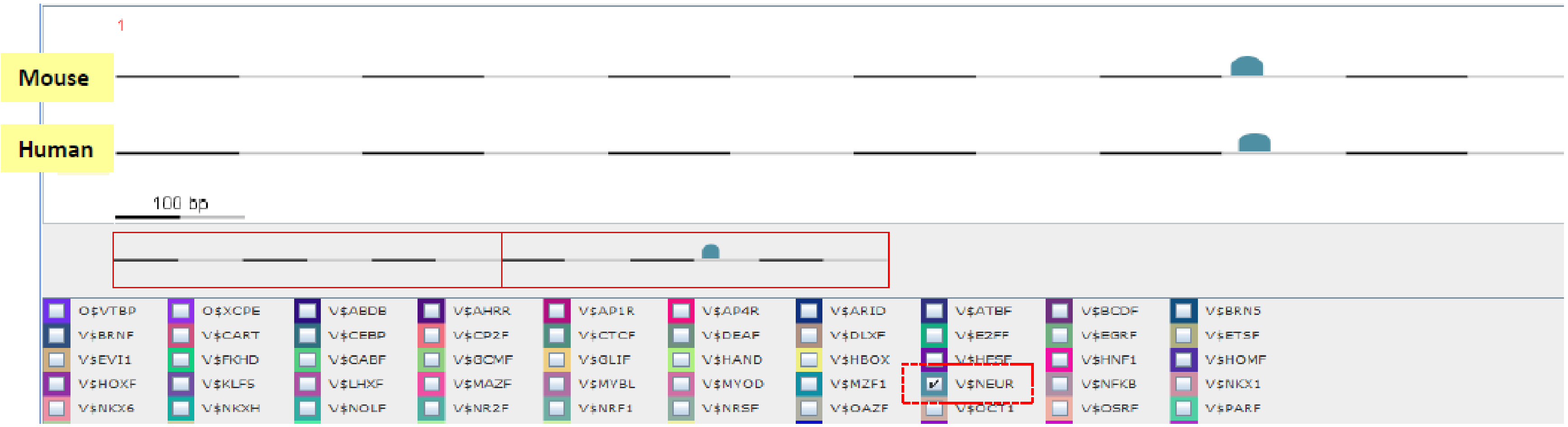

E-box

```
Human          TGCGGAGCGT CTGGAGCGGA GCACGCGCTG TCAGCTGGTG AGCGCACtct
Mouse          TGCGGAGCGT CTGGAGCGGA GCACGCGCTG TCAGCTGGTG AGCGCACtcg
Chick          TGCGCCGTGT CTGGAGTGGA GCACGCGCTG TCAGCTGGTG AGCGCggcgc
Zebra finch    TGCGCCGTGT CTGGAGCGGA GCACGCGTTG TCAGCTGGTG AGCGCA----
```

d)

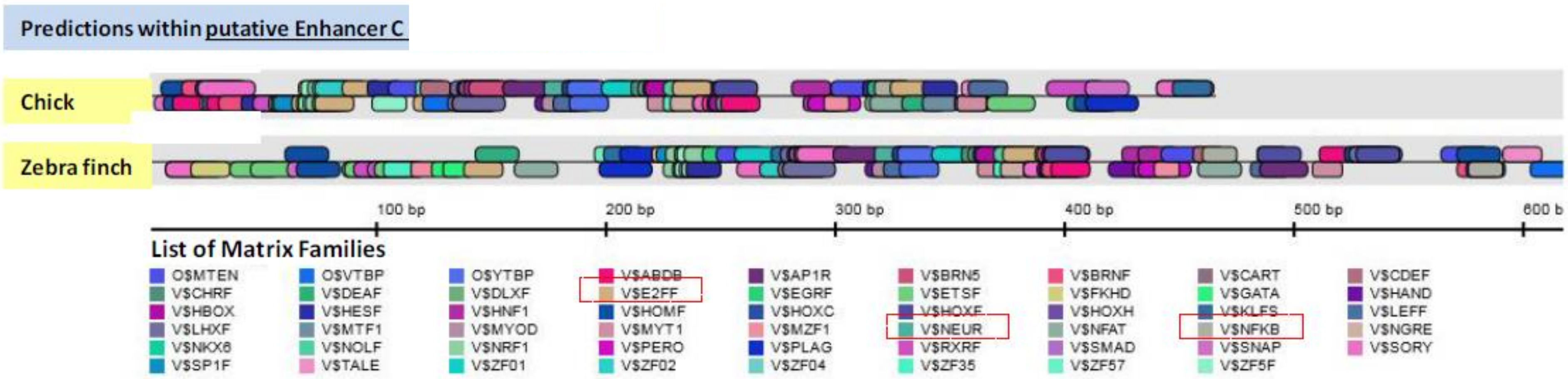
Predictions within putative Enhancer C
Chick
Zebra finch
100 bp
200 bp
300 bp
400 bp
500 bp
List of Matrix Families
O$MTEN
O$VTBP
O$YTBP
V$ABDB
V$AP1R
V$BRN5
V$BRNF
V$CART
V$CDEF
V$CHRF
V$DEAF
V$DLXF
V$E2FF
V$EGRF
V$ETSF
V$FKHD
V$GATA
V$HAND
V$HBOX
V$HESF
V$HNF1
V$HOMF
V$HOXC
V$HOXF
V$HOXH
V$KLFS
V$LEFF
V$LHXF
V$MTF1
V$MYOD
V$MYT1
V$MZF1
V$NEUR
V$NFAT
V$NFKB
V$NGRE
V$NKX6
V$NOLF
V$NRF1
V$PERO
V$PLAG
V$RXRF
V$SMAD
V$SNAP
V$SORY
V$SP1F
V$TALE
V$ZF01
V$ZF02
V$ZF04
V$ZF35
V$ZF57
V$ZF5F
