# Supplementary material for: Differential regulation of mammalian and avian ATOH1 by E2F1 and its implication for hair cell regeneration in the inner ear
Source: Sci Rep. 2021 Sep 29;11:19368. doi: 10.1038/s41598-021-98816-w (PMC8481459; doi:10.1038/s41598-021-98816-w)
Supplement: Supplementary file 4 — Supplementary Figure S3. [file 41598_2021_98816_MOESM4_ESM.pdf]

Differential regulation of mammalian and avian *ATOH1* by E2F1 and its implication for hair cell regeneration in the inner ear

Miriam Gómez-Dorado[1], Nicolas Daudet[1], Jonathan E. Gale[1], Sally J. Dawson[1*]

[1]UCL Ear Institute, 332 Gray's Inn Road, London WC1X 8EE, UK

Supplementary Figure S3. **Endogenous expression of E2F1 in the chick inner ear.** E2F1 showed faint levels of expression at E4 in the otocyst. At this stage early ATOH1 expression (in green) is observed in the anterior pro-sensory domain labelled with the prosensory marker SOX2 (arrows in the high magnification picture at E4). At E7, HCs marked with ATOH1 and SCs labelled with SOX2 have already emerged and E2F1 expression is ubiquitous in both the cochlear organ (basilar papilla) and in the vestibular system. A similar pattern of E2F1 expression is shown at E11 where E2F1 still appears ubiquitous in the basilar papilla and vestibular organs. At E18, E2F1 is strongly expressed in the nucleus of HCs and with less intensity in SCs (labelled with SOX2). Expression data are representative and collected from more than 4 animals (n >8 ears) for each developmental stage. Abbreviations: (ed) endolymphatic duct; (apd) anterior pro-sensory domain; (pc) posterior crista.

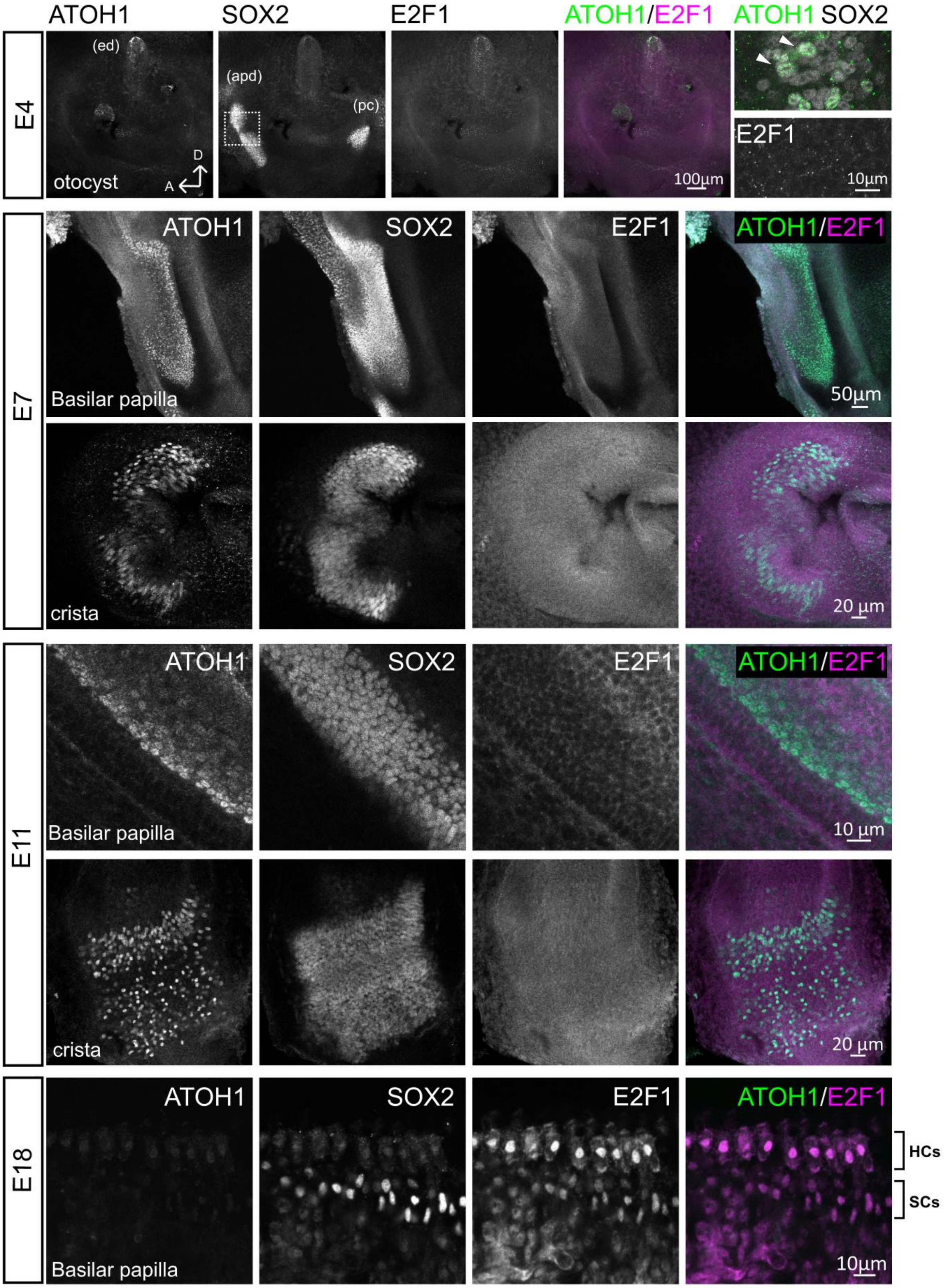
ATOH1
SOX2
E2F1
ATOH1/E2F1
ATOH1 SOX2
E4
(ed)
(apd)
(pc)
otocyst
D
A
100µm
E2F1
10µm
E7
Basilar papilla
50µm
crista
20 µm
E11
10 µm
E18
HCs
SCs
10µm
