# Supplementary material for: Differential regulation of mammalian and avian ATOH1 by E2F1 and its implication for hair cell regeneration in the inner ear
Source: Sci Rep. 2021 Sep 29;11:19368. doi: 10.1038/s41598-021-98816-w (PMC8481459; doi:10.1038/s41598-021-98816-w)
Supplement: Supplementary file 5 — Supplementary Figure S4. [file 41598_2021_98816_MOESM5_ESM.pdf]

Differential regulation of mammalian and avian *ATOH1* by E2F1 and its implication for hair cell regeneration in the inner ear

Miriam Gómez-Dorado[1], Nicolas Daudet[1], Jonathan E. Gale[1], Sally J. Dawson[1]*

[1]UCL Ear Institute, 332 Gray's Inn Road, London WC1X 8EE, UK

Supplementary Figure S4. Partial homology to a 268bp sequence of Chick Enhancer C within Mouse and Human Sequences and E2F binding site sequence alignments.

Legend:
***A*,** shows the sequence and location of the regions used in the Chick, Zebra finch, Human and Mouse sequence alignments used in alignments in **B**,**C**,**D** and **E** showing partial homology to a 286bp region of the enhancer C. All sequences are derived from Ensembl release 101 - August 2020. For all: the region highlighted in yellow corresponds to the chick 377bp Enh C which is the sequence cloned into the luc reporters; region highlighted in green corresponds to the E2F binding site within Enh C; base pairs marked in red corresponds to the core of the E2F S6 binding site within Enh C and those modified by site directed mutagenesis in luciferase experiments, this sequence is absent from human and mouse sequences.

<u>*A.* Chick Enhancer C Locus</u>

>4 dna:chromosome chromosome:GRCg6a:4:37047205:37047618:1
TTAATCGCTGTAATCCCCGTGTCCTCTCGCCCGCCCTGGTGCGCGCTCCCGCGCCCAACG
CGGGACAGCGACGCGCACCCGAGCGGTGCTGCCGCGCTTTATGGAGCGGTTAATCAACTG
CGCATCAGCGAGACAGCGCATCAGCCCATCTGCTTGATATATATTCAGGAGGGCTCCAGC
CCTTTTGAAGTCTAATTTCTTCCCCGGGAGAACGCGCCGGGTAATTTACCATCATTTCAT
ACGCATCGCGGCCACCGAGTTAACCCTTTCCCCAAGCGCTGCCCGCACGGGATTTTCCTC
CCGAAAAACGCCGGGTGCAACGCTACAAACTTGTCAAAGCGTCTTTCCGATGATGCTCCT
TGAAATAAGAGATCGCAGCTCACCGACACTGTAACCCCTTGGATCTGTCGGGTT

<u>Zebra finch</u>

>taeniopygia_guttata/1-498
TCCCG------------GTGTCTTGCGGCGGCCATCCCTTT-TCCCCTGCTCAACACCGA
GGCTCGCTGCCGCCGCCGGGGTGCCCGGAGAGAGGCGAGCGGCGGCGCGCACCCGCCGTG
GGCGAGCGGGGCTGCAGCACTTTTGTGGGGCGGCTAATCAATGGCACATCAGCGAGACAG
CGCATCAGCCCATCTGCTTGATATATATTCAGAAGGGCTCCAGCCCTTTTGAAGTCTAAT
TTCTTCCCCGGGAGAACGCGCCGGATAATTTACCATCATTTCCCAGCCAGGGCGGCCAGC
GGGTTAACCCTTTCCCCAAGCGCTGCCGTCGCGGG-TTTTCCTCCCGAGCGAGCAGCTTT
GAATGCCTCAGCGCCTCACTACAAACTTGTAAAAGTGCCTCTTTAATGGTACGTGACTCG
CTGAAACAGGACACC-CGGCCCACCGGCACGGTAAATCCCTCTCCGGCTTGTCAGATT--
-------GGTCTTTAAAA

<u>Human</u>

>4 dna:chromosome chromosome:GRCh38:4:93841005:93841300:1
TTTTTTCTCCCCTATTCTTGCTTCCCTCCCCTCTCTCTCCTGACAGAGACACCCAGCAGT
GTTTTGGTGGCTGTGCTGTCGACTTTCGCCTCCTAATCTCAGTTAATCAACTTGAGATCA
TTGATGCAGGTCAGCATCAGCCCATCTGTTTGATCAAGAGTCAGAGATCTCTATTTCCTT
GCAGTCAGAATTCCTCCCTGGGGCAGGCACCCTGCTATAGTAACCTTCATTTAATACCCC
CGTTATCATTAGCATCAGAGCTCATCGACACGCCTTTCCACAGAGGATCCCTGCTC

Mouse

```
>6 dna:chromosome chromosome:GRCm39:6:64718368:64718673:1
ATTGTGAACAGAATGCCTTTTCTCCTCCGTTCTTTGTTCTTTTCCTCCACTCCTCTGACA
AAGATACCCAGAATCTTGCTGTACCCTTTAGCTTTCTCTACTTAGTCTCTGGTAATCAGC
TGGGGATCCACAGGTCAGCATTGGCCCATCTATTTGATCAAGTGTTAGAGGTCTCTATTT
CTTTGTGGTTAGAATTCCTCCCAGGATAGACACCTGCTATAGTAACCTTCATTCAATGTC
CATTTATCTGTTGCTGGCATCAGAGCTCACCAACACTGTTCTTCCTCAGATTCCAGAGTC
AGCCTG
```

B. Pairwise Sequence Alignment of Enhancer C sequence in chick and zebra finch using EMBOSS Needle

Matrix: EBLOSUM62
Gap_penalty: 10.0
Extend_penalty: 0.5
Length: 477
Identity: 326/477 (68.3%)
Similarity: 326/477 (68.3%)
Gaps: 66/477 (13.8%)
Score: 1880.5

```
Chick_4            1 TTAATCGCTGTAATC-------------CCCGTGTCCTCTCGC-C--CGC     34
                         ||.|.|| .||             |||.|.|||.|| || |  |.|
Zebra_finch_t      1 ----TCCCGGT-GTCTTGCGGCGGCCATCCCTTTTCCCCT-GCTCAACAC     44

Chick_4           35 CCTGGTGCGC-GCTC-CCGC------GCCC-----AACGCGGGACAGCGA     71
                     |.:||..||| || | ||||      ||||     .|.|||.| |.|||.
Zebra_finch_t     45 CGAGGCTCGCTGC-CGCCGCCGGGGTGCCCGGAGAGAGGCGAG-CGGCGG     92

Chick_4           72 CGCGCACC---------CGAGCGGTGCTGCCGCGCTTTA-TGGAGCGGTT    111
                     ||||||||         |||||||.|||||.||.||||: |||.||||.|
Zebra_finch_t     93 CGCGCACCCGCCGTGGGCGAGCGGGGCTGCAGCACTTTTGTGGGGCGGCT    142

Chick_4          112 AATCAACTGCGCATCAGCGAGACAGCGCATCAGCCCATCTGCTTGATATA    161
                     ||||||..||.|||||||||||||||||||||||||||||||||||||||
Zebra_finch_t    143 AATCAATGGCACATCAGCGAGACAGCGCATCAGCCCATCTGCTTGATATA    192

Chick_4          162 TATTCAGGAGGGCTCCAGCCCTTTTGAAGTCTAATTTCTTCCCCGGGAGA    211
                     |||||||.||||||||||||||||||||||||||||||||||||||||||
Zebra_finch_t    193 TATTCAGAAGGGCTCCAGCCCTTTTGAAGTCTAATTTCTTCCCCGGGAGA    242

Chick_4          212 ACGCGCCGGGTAATTTACCATCATTTCATACGCATC----GCGGCCACCG    257
                     |||||||||.|||||||||||||||||   | ||.|    |||||||.||
Zebra_finch_t    243 ACGCGCCGGATAATTTACCATCATTTC---C-CAGCCAGGGCGGCCAGCG    288

Chick_4          258 AGTTAACCCTTTCCCCAAGCGCTGCCCGCACGGGATTTTCCTCCCGAA-A    306
                     .|||||||||||||||||||||||||..|.|||| ||||||||||||. .
Zebra_finch_t    289 GGTTAACCCTTTCCCCAAGCGCTGCCGTCGCGGG-TTTTCCTCCCGAGCG    337

Chick_4          307 AAC----------GCCG-GGTGCAACGCTACAAACTTGTCAAA--GCGTC    343
                     |.|          |||. .|.||.:|.||||||||||||.|||  ||.||
Zebra_finch_t    338 AGCAGCTTTGAATGCCTCAGCGCCTCACTACAAACTTGTAAAAGTGCCTC    387

Chick_4          344 TT------TCCGATGATGCTC-CTTGAAATAAGAGATCGCAGCTCACCGA    386
                     ||      |.|| |||  ||| | |||||.|.||.|.| |.||.|||||.
Zebra_finch_t    388 TTTAATGGTACG-TGA--CTCGC-TGAAACAGGACACC-CGGCCCACCGG    432

Chick_4          387 CACTGTAA--CCC-CT-TGGATC-TGTCGGGTT-----------    414
                     |||.||||  ||| || .||  | ||||.|.||
Zebra_finch_t    433 CACGGTAAATCCCTCTCCGG--CTTGTCAGATTGGTCTTTAAAA    474
```

C. Pairwise Sequence Alignment of Enh C sequence in chick and human using EMBOSS Needle

```
# Matrix: EBLOSUM62
# Gap_penalty: 10.0
# Extend_penalty: 0.5
# Length: 437
# Identity:      191/437 (43.7%)
# Similarity:    191/437 (43.7%)
# Gaps:          164/437 (37.5%)
# Score: 935.5



Chick_4                  1 -TTAATCGCTGTAATCCCCGTGTCCTCTCGCCCGCCCTGGTGCGCGCTCC     49
                            ||..||       |||||...||.|....|||.|||         |||.
Human_4                  1 TTTTTTC-------TCCCCTATTCTTGCTTCCCTCCC---------CTCT     34

Chick_4                 50 CGCGCCCAACGCGGGACAGCGACGCGCACCCG---------AGCGGTGCT     90
                           |.|.||.       |||||.|||.|.||.|.|         .||.|||||
Human_4                 35 CTCTCCT-------GACAGAGACACCCAGCAGTGTTTTGGTGGCTGTGCT     77

Chick_4                 91 GCCG-----CGCTTTATGG-AGCGGTTAATCAACTGCGCATCAGCGAGAC    134
                           |.||     |||.|..|.. ..|.|||||||||||....||||..||..|
Human_4                 78 GTCGACTTTCGCCTCCTAATCTCAGTTAATCAACTTGAGATCATTGATGC    127

Chick_4                135 AG---CGCATCAGCCCATCTGCTTGATATATATTCAGGAGGGCTCCA--G    179
                           ||   .|||||||||||||||.|||||..|.|.||| |||..|||.|  .
Human_4                128 AGGTCAGCATCAGCCCATCTGTTTGATCAAGAGTCA-GAGATCTCTATTT    176

Chick_4                180 CCCTTTTGAAGTCTAATTTCTTCCCCGGGAGAACGCGCCGGGTAAT--TT    227
                           ||   |||.||||..|.|||.||||.||| |.|.||.||..|..||  |.
Human_4                177 CC---TTGCAGTCAGAATTCCTCCCTGGG-GCAGGCACCCTGCTATAGTA    222

Chick_4                228 ACCATCATTTCATACGCATCGCGGCCACCGAGTTAACCCTTTCCCCAAGC    277
                           |||.||||||.||||         ||.|   ||||.|..|..|..| ||.
Human_4                223 ACCTTCATTTAATAC---------CCCC---GTTATCATTAGCATC-AGA    259

Chick_4                278 GCTGCCCGCACGGGATTTTCCTCCCGAAAAACGCCGGGTGCAACGCTACA    327
                           |||                                     ||.||  |||
Human_4                260 GCT-------------------------------------CATCG--ACA    270

Chick_4                328 AACTTGTCAAAGCGTCTTTCCGATGATGCTCCTTGAAATAAGAGATCGCA    377
                                        ||.||||||...||.|.|||               |.
Human_4                271 -------------CGCCTTTCCACAGAGGATCC---------------CT    292

Chick_4                378 GCTCACCGACACTGTAACCCCTTGGATCTGTCGGGTT    414
                           ||||
Human_4                293 GCTC---------------------------------    296
```

D. Pairwise Sequence Alignment of Enh C sequence in chick and mouse using EMBOSS Needle

Matrix: EBLOSUM62
Gap_penalty: 10.0
Extend_penalty: 0.5
Length: 429
Identity: 187/429 (43.6%)
Similarity: 187/429 (43.6%)
Gaps: 138/429 (32.2%)
Score: 840.0

```
Chick_4            1 -TTAATCGCTGTAATCCCCGTGTCCTCTCGCCCGCCCTGGTGCGCGCTCC     49
                      ||.....|.|.|..||     |..||||..|||..|| .||..|..|.|
Mouse_6            1 ATTGTGAACAGAATGCC-----TTTTCTCCTCCGTTCT-TTGTTCTTTTC     44

Chick_4           50 CGCGCCCAACGCGGGACAGCGACGCGCACCCGAGCGGTGCTG--CCGC--     95
                     |.| |.|..|.| .||||..||..|   ||.||....|||||  || |
Mouse_6           45 CTC-CACTCCTC-TGACAAAGATAC---CCAGAATCTTGCTGTACC-CTT     88

Chick_4           96 --GCTTTAT-------GGAGCGGTTAATCAACTGCGCATCAGCGAGACAG    136
                       |||||.|       |...|.|.||||||.|||.|.|||..|..|.||
Mouse_6           89 TAGCTTTCTCTACTTAGTCTCTGGTAATCAGCTGGGGATCCACAGGTCA-    137

Chick_4          137 CGCATCAGCCCATCTGCTTGAT-ATATATTCAGGAGGGCTCCAGCCCTTT    185
                      ||||..||||||||..||||| |..|.||  .||||.|||.|...| ||
Mouse_6          138 -GCATTGGCCCATCTATTTGATCAAGTGTT--AGAGGTCTCTATTTC-TT    183

Chick_4          186 TGAAGTCTAATTTCTTCCCCGGGAGAACGCGCCGGGTAATTTACCATCAT    235
                     ||..||...|.|||.||||.||....||.| |.|....|.|.|||.||||
Mouse_6          184 TGTGGTTAGAATTCCTCCCAGGATAGACAC-CTGCTATAGTAACCTTCAT    232

Chick_4          236 TTCATACGCATCGCGGCCACCGAGTTAACCCTTTCCCCAAGCGCTGCCCG    285
                     |..||         |.|||     ||.|.|..||        ||||.|..
Mouse_6          233 TCAAT---------GTCCA-----TTTATCTGTT--------GCTGGCAT    260

Chick_4          286 CACGGGATTTTCCTCCCGAAAAACGCCGGGTGCAACGCTACAAACTTGTC    335
                     ||..|       |||.|               ||||.||
Mouse_6          261 CAGAG-------CTCAC---------------CAACACT-----------    277

Chick_4          336 AAAGCGTCTTTCCGATGATGCTCCTTGAAATAAGAGATCGCAGCTCACCG    385
                          ||..||||...|||  |||        ||||.   ||||
Mouse_6          278 -----GTTCTTCCTCAGAT--TCC--------AGAGT---CAGC------    303

Chick_4          386 ACACTGTAACCCCTTGGATCTGTCGGGTT    414
                        |||
Mouse_6          304 ---CTG-----------------------    306
```

E. Cross-species alignment of E2F binding site S6 using MatInspector, Genomatix Platform (as described in Figure 4 of the main manuscript).

```
Chick          GCGCCCAACGCGGG
Zebra finch    GCCGCCGGGGTGCC
Human          TCTCC---------------TG
Mouse          TCCTC---------------TG
```
