# Supplementary material for: Differential regulation of mammalian and avian ATOH1 by E2F1 and its implication for hair cell regeneration in the inner ear
Source: Sci Rep. 2021 Sep 29;11:19368. doi: 10.1038/s41598-021-98816-w (PMC8481459; doi:10.1038/s41598-021-98816-w)
Supplement: Supplementary file 6 — Supplementary Table S1. [file 41598_2021_98816_MOESM6_ESM.pdf]

Differential regulation of mammalian and avian *ATOH1* by E2F1 and its implication for hair cell regeneration in the inner ear

Miriam Gómez-Dorado[1], Nicolas Daudet[1], Jonathan E. Gale[1], Sally J. Dawson[1]*

[1]UCL Ear Institute, 332 Gray's Inn Road, London WC1X 8EE, UK

Supplementary Table 1. EMSA oligonucleotides containing the putative E2F sites in the *Atoh1* regulatory regions. Each probe contains an E2F binding site predicted by the Genomatix analysis. The predicted E2F binding sites are underlined in each probe. Probes were referred to as probe 1-9 corresponding to the nine E2F sites predicted in the chick *Atoh1* regulatory elements.

Oligo name	Primer Sequence (5' to 3')	Predicted Binding site
**Probe 1_Sense**	AGCCGCGAAGTGCGCGCTCTCCGCTTTGC	E2F2 and E2F3
**Probe 1_Antisense**	GCAAAGCGGAGAGCGCGCACTTCGCGGCT	E2F2 and E2F3
**Probe 2_Sense**	GCCTCAAAAAGCGATAAAAAATGGCACA	E2F1&E2F4
**Probe 2_Antisense**	TGTGCCATTTTTTATCGCTTTTTGAGGC	E2F1&E2F4
**Probe 3_Sense**	TCCCGGCCGCGGTGCGCCGTGTCTGGA	E2F2 and E2F3
**Probe 3_Antisense**	TCCAGACACGGCGCACCGCGGCCGGGA	E2F2 and E2F3
**Probe 4_Sense**	CTTTCAGGCGGCGCCCCGGGGAGCTGC	E2F3
**Probe 4_Antisense**	GCAGCTCCCCGGGGCGCCGCCTGAAAG	E2F3
**Probe 5_Sense**	TGTCCTCTCGCCCGCCCTGGTGCGCGCTCCCGC	E2F1, E2F2 and E2F3
**Probe 5_Antisense**	GGAGCGCGCACCAGGGCGGGCGAGAGGACA	E2F1, E2F2 and E2F3
**Probe 6_Sense**	TGCGCGCTCCCGCGCCCAACGCGGGACAGCGACGCGC	E2F1 and E2F4
**Probe 6_Antsense**	GCGCGTCGCTGTCCCGCGTTGGGCGCGGGAGCGCGCA	E2F1 and E2F4
**Probe 7_Sense**	GAGCGGTGCTGCCGCGCTTTATGGAGC	E2F2
**Probe 7_Antisense**	GCTCCATAAAGCGCGGCAGCACCGCTC	E2F2
**Probe 8_Sense**	ATTTCTTCCCCGGGAGAACGCGCCGG	E2F1
**Probe 8_Antisense**	CCGGCGCGTTCTCCCGGGGAAGAAAT	E2F1
**Probe 9_Sense**	GATTTTCCTCCCGAAAAACGCCGGGT	E2F1
**Probe 9_Antisense**	ACCCGGCGTTTTTCGGGAGGAAAATC	E2F1
